# Experimental Cerebral Malaria Pathogenesis—Hemodynamics at the Blood Brain Barrier

**DOI:** 10.1371/journal.ppat.1004528

**Published:** 2014-12-04

**Authors:** Adéla Nacer, Alexandru Movila, Fabien Sohet, Natasha M. Girgis, Uma Mahesh Gundra, P'ng Loke, Richard Daneman, Ute Frevert

**Affiliations:** 1 Department of Microbiology, Division of Medical Parasitology, New York University School of Medicine, New York, New York, United States of America; 2 Department of Anatomy, University of California San Francisco, San Francisco, California, United States of America; Case Western Reserve University, United States of America

## Abstract

Cerebral malaria claims the lives of over 600,000 African children every year. To better understand the pathogenesis of this devastating disease, we compared the cellular dynamics in the cortical microvasculature between two infection models, *Plasmodium berghei* ANKA (PbA) infected CBA/CaJ mice, which develop experimental cerebral malaria (ECM), and *P. yoelii* 17XL (PyXL) infected mice, which succumb to malarial hyperparasitemia without neurological impairment. Using a combination of intravital imaging and flow cytometry, we show that significantly more CD8+ T cells, neutrophils, and macrophages are recruited to postcapillary venules during ECM compared to hyperparasitemia. ECM correlated with ICAM-1 upregulation on macrophages, while vascular endothelia upregulated ICAM-1 during ECM and hyperparasitemia. The arrest of large numbers of leukocytes in postcapillary and larger venules caused microrheological alterations that significantly restricted the venous blood flow. Treatment with FTY720, which inhibits vascular leakage, neurological signs, and death from ECM, prevented the recruitment of a subpopulation of CD45^hi^ CD8+ T cells, ICAM-1+ macrophages, and neutrophils to postcapillary venules. FTY720 had no effect on the ECM-associated expression of the pattern recognition receptor CD14 in postcapillary venules suggesting that endothelial activation is insufficient to cause vascular pathology. Expression of the endothelial tight junction proteins claudin-5, occludin, and ZO-1 in the cerebral cortex and cerebellum of PbA-infected mice with ECM was unaltered compared to FTY720-treated PbA-infected mice or PyXL-infected mice with hyperparasitemia. Thus, blood brain barrier opening does not involve endothelial injury and is likely reversible, consistent with the rapid recovery of many patients with CM. We conclude that the ECM-associated recruitment of large numbers of activated leukocytes, in particular CD8+ T cells and ICAM+ macrophages, causes a severe restriction in the venous blood efflux from the brain, which exacerbates the vasogenic edema and increases the intracranial pressure. Thus, death from ECM could potentially occur as a consequence of intracranial hypertension.

## Author Summary

Malaria remains one of the most serious health problems globally, but our understanding of the biology of the *Plasmodium* parasite and the pathogenesis of severe disease is still limited. Human cerebral malaria (HCM), a severe neurological complication characterized by rapid progression from headache to convulsions and unrousable coma, causes the death of hundreds of thousands of children in Africa annually. To better understand the pathogenesis of cerebral malaria, we imaged immune cells in brain microvessels of mice with experimental cerebral malaria (ECM) versus mice with malarial hyperparasitemia, which lack neurological impairment. Death from ECM closely correlated with plasma leakage, platelet marginalization, and the recruitment of significantly more leukocytes to postcapillary venules compared to hyperparasitemia. Leukocyte arrest in postcapillary venules caused a severe restriction in the venous blood flow and the immunomodulatory drug FTY720 prevents this recruitment and death from ECM. We propose a model for ECM in which leukocyte arrest, analogous to the sequestration of *P. falciparum* infected red blood cells in HCM, severely restricts the venous blood flow, which exacerbates edema and swelling of the brain at the agonal comatose stage of the infection, leading to intracranial hypertension and death.

## Introduction

*Plasmodium falciparum* is responsible for an estimated 600,000 deaths annually, principally in children under the age of five [1]. Clinical symptoms range from intermittent fevers and chills to potentially fatal complications including severe anemia and cerebral malaria [2]. The mortality rate in comatose pediatric patients, most frequently due to respiratory arrest, is 15–20% despite optimal medical care [3], but the underlying pathology is unclear.

Molecular and cellular mechanisms involved in the pathogenesis of human cerebral malaria (HCM) include a predominantly pro-inflammatory cytokine profile, endothelial activation via the NF-κB pathway with upregulation of adhesion molecules, glia cell activation, and sequestration of infected red blood cells (iRBC), monocytes, and platelets within brain capillaries [3]–[6]. However, the cellular mechanisms associated with HCM cannot be directly observed in the human brain. Ophthalmological examination of the retinal pathology generally correlates with course and etiology of malarial encephalopathy [2], [7], but despite significant recent improvements [8], this technique lacks the resolution to observe the dynamic behavior of individual iRBC, leukocytes, and platelets, their exact location within the microvasculature, mechanisms of vascular leakage or possibly occlusion, and the sequence of these events. Elucidation of CM pathogenesis therefore requires the use of a robust small animal model that closely reflects clinical symptoms, histopathology, and immune mechanisms associated with the pathophysiology of HCM.

*P. berghei* ANKA (PbA) infected CBA, Swiss Webster, or CB57Bl/6 mice represent a well-characterized and widely used model for experimental cerebral malaria (ECM) that shares a number of similarities with *P. falciparum* HCM [5], [6], [9]–[12]. Both ECM and HCM are characterized by severe vasculopathy, i.e. endothelial activation and dysfunction with increased expression of adhesion molecules such as ICAM-1, VCAM-1, and E-selectin, upregulation of inflammatory cytokines, reduced blood flow, vascular leakage, acute edema of both vasogenic and cytotoxic origin, and microhemorrhages leading to neurological impairment [12]–[17]. Platelet activation, dysregulation of the coagulation cascade, thrombocytopenia, and platelet accumulation in the brain are also found in both HCM and ECM [18]–[20]. We have previously shown by intravital microscopy (IVM) that platelet marginalization and blood brain barrier (BBB) disruption are central to ECM pathophysiology [21]. Platelets are thought to impair vascular repair and increase BBB permeability by potentiating the iRBC-induced endothelial damage in the early stages of HCM development [22]–[24]. Circulating platelet-derived microparticles are increased in severe *P. falciparum* malaria and serve as a biomarker for neurological involvement [13], [25].

The murine PbA model has also provided ample evidence for a contribution of CD8+ and CD4+ T cells to the late stages of ECM development [26]–[30]. Both CD8+ T cells, generally considered the terminal effector cells, and CD4+ T cells must accumulate in the cerebral microvasculature for ECM to occur [11], [27], [28], [31]–[35] and may also be responsible for the ECM-associated leukocyte infiltration [36]. While ECM development was thought to involve CD8+ T cell-induced endothelial apoptosis via perforin- and granzyme B-mediated cytotoxicity resulting in BBB disruption [32], [33], [37], we recently showed by IVM that ECM closely correlates with widespread opening of the BBB and that this occurs in the absence of significant endothelial death [21]. The BBB at the level of postcapillary venules encompasses two layers, the vascular endothelium with its basement membrane and the glia limitans with associated basement membranes and astrocyte endfeet, which are separated by the perivascular space [38]. This section of the BBB is functionally distinct from other areas of the BBB, for example that at the capillary level, which consists of a single layer composed of endothelia, gliovascular membrane, and astrocyte endfeet [38]. IVM also revealed that ECM correlates with platelet deposition, leukocyte arrest, and *de novo* expression of the pattern recognition receptor CD14 on the endothelial surface from postcapillary venules, but not from capillaries or arterioles [21]. Strikingly, inhibition of platelet deposition and leukocyte recruitment by blockage of LFA-1 mediated cellular interactions prevented ECM and disruption of the BBB in PbA-infected mice [21]. Thus, it appears that the ultimate cause of coma and death in ECM is a universal breakdown of the BBB at the level of postcapillary venules [21].

In the PbA-infected CBA/CaJ mouse model, vascular leakage, neurological signs, and death from ECM can be prevented by treatment with the endothelial barrier-stabilizing sphingosine 1 analog FTY720 (fingolimod) [21], [39], an immunomodulatory FDA-approved drug for oral treatment of relapsing multiple sclerosis (MS) [40] that acts as an agonist for sphingosine 1-phosphate (S1P) receptors [41]. In experimental autoimmune encephalomyelitis (EAE), FTY720 prevents T cell recruitment to the brain by down-modulating the expression of S1P1 receptors on the T cell surface. This favors the CCR7-mediated retention of naïve and central memory T cells within secondary lymphatic tissues [42], leading to a reduction in the numbers of naïve and central memory T cells, but not effector memory T cells, in the blood [43]. FTY720 may also prevent stimulation of vascular endothelia or activation of CD8+ effector T cells in the spleen by decreasing CD11c+ DC migration and function and by destabilizing DC/T cell interactions thus preventing the formation of an immunological synapse [44], [45]. In addition to its involvement in T cell activation and targeting to the brain, FTY720 is also thought to have a directly stabilizing effect on endothelial junctions at the BBB [46]–[49]. However, the exact mechanism by which FTY720 prevents BBB opening remains unclear to date.

Here, we show that ECM is associated with the accumulation of numerous leukocytes within postcapillary and larger venules and that the resulting microrheological alterations severely restrict the venous blood flow. Treatment with FTY720 significantly reduced the recruitment of these leukocytes indicating their involvement in the pathogenesis of ECM [21], [39]. Leukocyte arrest likely increases the intracranial pressure, similarly to *P. falciparum* iRBC sequestration in pediatric HCM, which is typically associated with a poor clinical outcome [50].

## Results

Three week-old CBA/CaJ mice were infected with PbA-GFP or *P. yoelii* 17XL (PyXL)-RFP and monitored throughout the course of development of ECM or hyperparasitemia [21]. In this study, a total of 78 PbA-infected mice were subjected to IVM at the time of ECM (day 6–8), 18 PbA-infected mouse before ECM (day 5), 15 PbA-infected mice that failed to develop ECM (day 9), and 25 uninfected control mice (**Table S1**). We also examined 30 PbA-infected mice that were treated with FTY720 and failed to develop ECM (day 8–9) and 8 PbA-infected mice that developed ECM on day 8 despite treatment with FTY720. As controls for parasitemia, 62 PyXL-infected mice with >50% parasitemia (day 5) were analyzed. Throughout our IVM experiments, we examined deep microvessels, i.e. branches of penetrating arterioles and venules [51], [52], which were in direct continuation with the cortical capillary bed. Imaging of CX3CR1^GFP/+^ mice confirmed that the postcapillary venules, capillaries, and arterioles used for analysis are embedded in fluorescent microglia and thus clearly located in the cerebral cortex, i.e. underneath the pial microvasculature (**Figure S1**) [21].

### ECM correlates with microrheological alterations in postcapillary venules

**Figure 1:**
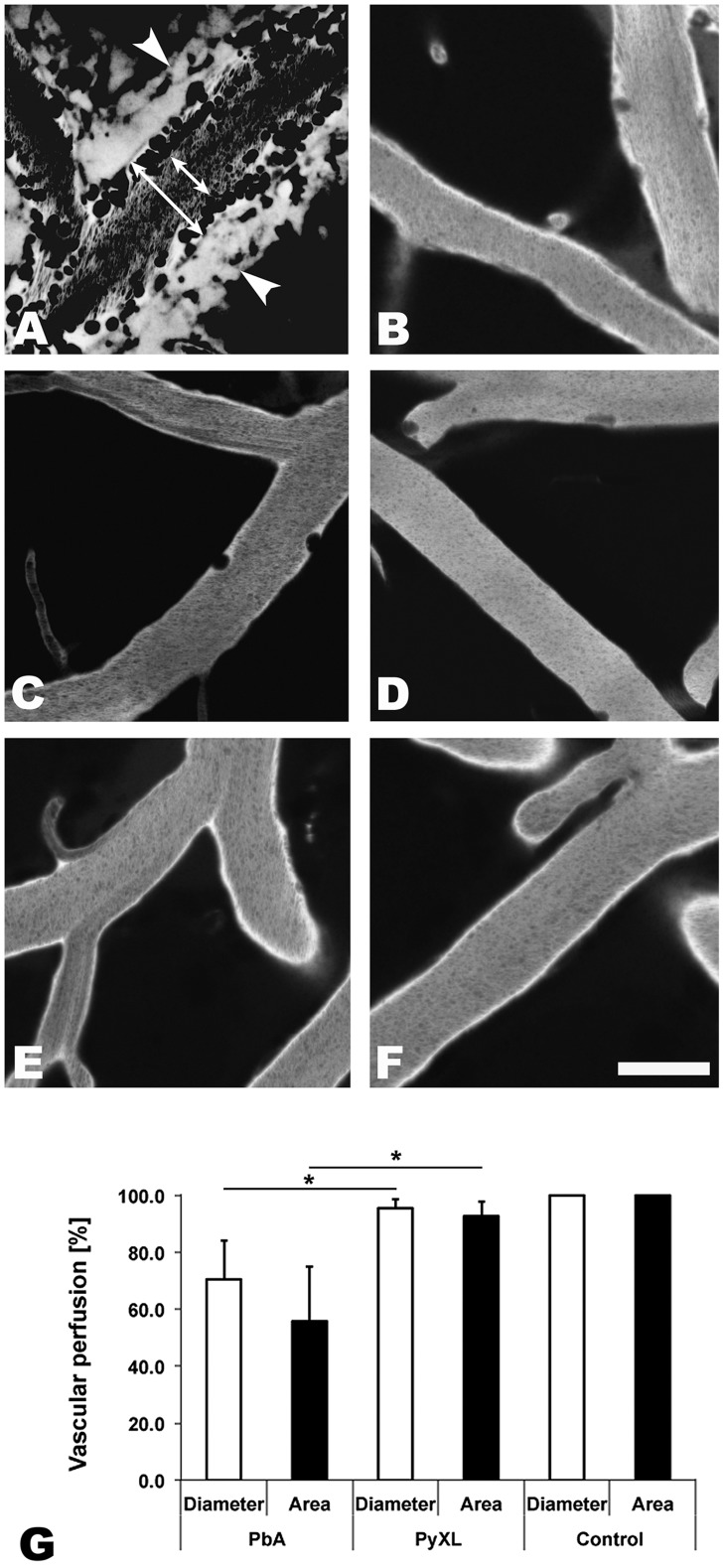
ECM correlates with microrheological alterations in postcapillary venules. CBA/CaJ mice were infected with PbA, PyXL, or no parasites. To assess the blood flow within the cortical microvasculature, time sequences were converted to minimal projections. **A**) In mice with ECM, the functional postcapillary venule diameter (short arrow), i.e. the perfused portion of the vessel, is considerably reduced compared to the entire vessel diameter (long arrow). Visualization of the vascular lumen with Evans blue reveals a zone along the endothelium of postcapillary venules (**A**) that contains adherent leukocytes (dark circles or ovals), but is devoid of RBC (dark streaks in the center). Note that migrating leukocytes are represented multiple times in minimal projections. Leakage of Evans blue into the perivascular space and brain parenchyma is apparent on either side of the postcapillary venule (arrowheads). **B**) Arterioles from mice with symptomatic ECM do not exhibit any restriction in diameter. **C**) The small number of adherent leukocytes (dark circles) in mice with hyperparasitemia does not cause any significant restriction in the functional postcapillary venule lumen. Neither arterioles from mice with hyperparasitemia (**D**) nor postcapillary venules or arterioles from uninfected control mice (**E and F**) exhibit any microrheological alterations. Scale bars = 20 µm. See **Videos S1–S5** for the corresponding dynamic data. **G**) Measurement of the total and functional vascular diameters and cross-sections reveals that the blood flow in postcapillary venules from mice with ECM, but not from mice with hyperparasitemia, is severely restricted. Postcapillary venules from uninfected control mice exhibit no luminal restriction.

IVM revealed that the venous blood flow in postcapillary venules from PbA-infected mice with neurological signs (day 6) was strikingly altered. Vascular labeling with Evans blue revealed that postcapillary venules from mice with ECM exhibited a marginal zone devoid of RBCs (**Figure 1A** **, Video S1**). Instead, this zone contained variable numbers of leukocytes that were either rolling along the endothelium, crawling, or firmly attached (**Figure S2A and S2C, Video S2**). Minimal projections of time sequences emphasize the boundary between the functional lumen in the center of the postcapillary venules and the RBC-free marginal zone and suggest that the functional lumen available for the blood flow is significantly restricted during ECM (**Figure S2B and S2D**). This phenomenon was even more pronounced in larger venules. Neither arterioles from mice with ECM (**Figure 1B** **, Video S3**) nor postcapillary venules or arterioles from mice with hyperparasitemia (**Figure 1C and 1D** **, Video S4**) showed any significant functional vascular restriction, i.e. narrowing of the passageway available for the blood flow, compared to uninfected control mice (**Figure 1E and 1F** **, Video S5**), a finding we attribute to the absence of steric hindrance generated by adherent leukocytes in these vessels. Multiple measurements of the total vascular diameter (from endothelium to endothelium) and the functional diameter (used by the blood flow) of 50 randomly chosen postcapillary venules from 4 mice with ECM revealed a mean functional diameter of 70.5±13.7% compared to data from 3 uninfected control mice, corresponding to a functional vascular cross-section of 55.8±19.1% (**Figure 1G**). Notably, complete vascular occlusion, whether in postcapillary venules or other microvessels, was not observed during ECM. In 50 randomly chosen postcapillary venules from 3 PyXL-infected mice with hyperparasitemia, the functional postcapillary venule diameter and cross-section was 95.5±3.4% and 92.7±5.1%, respectively. No microrheological alterations were found in postcapillary venules or arterioles from PbA-infected mice prior to ECM (day 5), in PbA-infected mice that failed to develop ECM (day 9), or in uninfected control mice. As reported previously [21], PbA-infected mice that did develop ECM despite treatment FTY720 exhibited vascular leakage suggesting that the venous blood flow restriction was similar to untreated PbA-infected mice with ECM.

Thus, ECM correlates with a significant functional constraint, but not complete blockage, of the passageway available for the venous blood flow. This is significant, because any restriction in the venous efflux from the brain likely exacerbates edema formation, a hallmark of both ECM and HCM [16], [17], [53]. A venous efflux problem would also explain the increased intracranial pressure, which is frequently observed in pediatric CM in Africa [50]. Indeed, MRI imaging has identified increased intracranial pressure as the strongest predictor of death [54], [55].

### Leukocyte recruitment during ECM and hyperparasitemia

**Figure 2:**
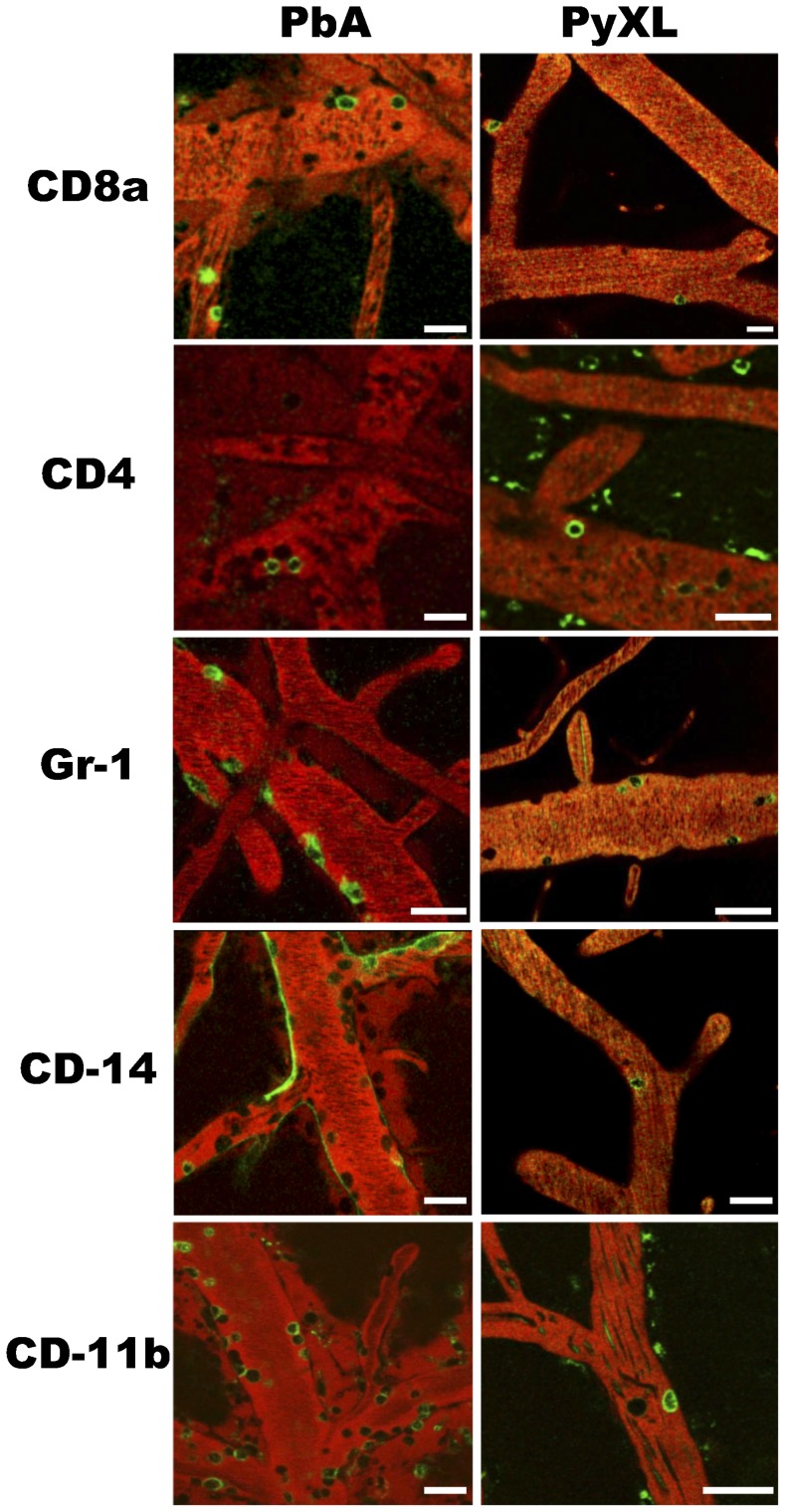
Leukocytes are recruited to cortical postcapillary venules during both ECM and hyperparasitemia. CBA/CaJ mice were infected with PbA, PyXL, or no parasites, subjected to craniotomy at the time of neurological signs or the parasitemia exceeding 50%, and prepared for intravital microscopy. CD8+ T cells, CD4+ T cells, neutrophils, monocytes, and macrophages were labeled by intravenous inoculation of fluorochrome-conjugated mAb (see Materials and Methods for details) and appear as open green circles. The vascular lumen was visualized with Evans blue (red). Representative images from intravital microscopy movies showing arrested green CD8+ T cells (CD8a), CD4+ T cells (CD4), neutrophils (GR-1), monocytes (CD14), and macrophages (CD11b) in postcapillary venules from PbA-infected mice with ECM and PyXL-infected mice with hyperparasitemia. Note that anti-CD14 labels the endothelium (green outline) during ECM in addition to monocytes (open green circles). Scale bars = 20 µm. See **Videos S6–S16** for dynamic information.

**Figure 3:**
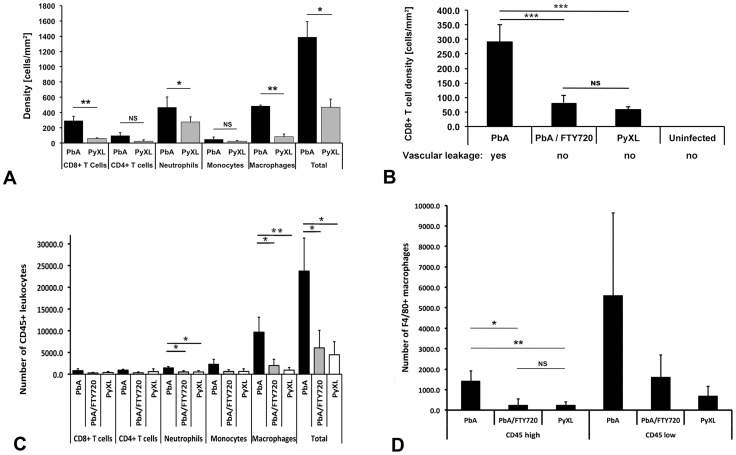
Leukocyte recruitment to cortical postcapillary venules during ECM and hyperparasitemia. **A**) CBA/CaJ mice were infected with PbA, PyXL, or no parasites, subjected to craniotomy at the time of neurological signs or the parasitemia exceeding 50%, and prepared for intravital microscopy. CD8+ T cells, CD4+ T cells, Gr-1+ neutrophils, CD14+ monocytes, and CD11b+ macrophages were labeled by intravenous inoculation of mAb against CD8+a, CD4+, GR-1, CD14, and CD11b, respectively. Significantly larger numbers of CD8+ T cells, neutrophils and macrophages were found in postcapillary venules from mice with ECM compared to hyperparasitemia (*P*<0.05). The density of CD4+ T cells and monocytes was not significantly different. Most of the arrested leukocytes are macrophages followed by neutrophils, CD8+ T cells, CD4+ T cells, and monocytes. The data represent the mean cell density/mm^2^ ± STD. Significance (*, *P*<0.05; **, *P*<0.001) was determined with 1-way ANOVA. See **Tables S2–S6** for details. **B**) Compared to mice with ECM (PbA), significantly less CD8+ T cells are recruited to postcapillary venules from mice with hyperparasitemia (PyXL). Treatment with FTY720 reduces the density of CD8+ T cells to levels similar to those found in PyXL infected mice. No arrested CD8+ T cells were found in the cortical microvasculature from uninfected mice. Vascular leakage was observed in mice with ECM (day 6–8; 76 measurements from 5 mice), but not in FTY720-treated PbA-infected mice (day 8; 19 measurements from 4 mice), PyXL infected mice with hyperparasitemia (day 5; 20 measurements from 6 mice), or uninfected control mice (>10 mice). **C**) Leukocytes were isolated from the brains of PbA-infected (day 6–8), PbA-infected/FTY720-treated (day 9), or PyXL-infected mice (day 5) and analyzed by flow cytometry. Most of the ECM-associated leukocytes are F4/80+ macrophages followed by Ly-6C+ monocytes, Ly-6G+ neutrophils, CD8+ T cells, and CD4+ T cells. Significance (*, *P*<0.05; **, *P*<0.01) was determined with 1-way ANOVA followed by Tukey's test for multiple comparisons. See **Table S7** for details. **D**) FTY720 treatment of PbA-infected mice reduces the number of CD45^hi^ ( = blood-derived) macrophages significantly to a level similar to that found in PyXL-infected mice with hyperparasitemia. No significant differences were found for CD45^lo^ ( = parenchymal) macrophages. See **Table S7** for details.

Quantitative offline IVM analysis of the various cell densities revealed that leukocytes are recruited to the cortical microvasculature not only in response to ECM, but also hyperparasitemia, albeit at a significantly lower density (**Figure 2**). Specifically, more CD8+ T cells, neutrophils, and macrophages were recruited in ECM compared to hyperparasitemia, while the density of all other cell types analyzed did not differ between these two infections (**Figure 3A**). The cortical microvasculature of uninfected control mice exhibited virtually no arrested leukocytes suggesting that in the absence of an inflammatory stimulus, innate immune cells do not monitor the BBB.

#### CD8+ and CD4+ T cells

On the day of neurological signs, the density of CD8+ T cells arrested in postcapillary venules from PbA-infected mice was 292.1±58.9 cells/mm^2^ (**Figure 3A**, **Table S2**). While 92.7% of these T cells were intravascular, 7.3% had extravasated into the perivascular space. No CD8+ T cells were found on the day prior to ECM or in mice that survived the critical period of ECM development. Interestingly, CD8+ T cells were also found in postcapillary venules from PyXL-infected mice with hyperparasitemia and severe anemia. However, with 61.0±8.1 cells/mm^2^, the density was significantly lower (t-test: T_(4)_ = 8.69; *P*<0.01) and no T cells were found in the perivascular space of these mice. CD8+ T cells were absent from the cortical microvasculature of uninfected control mice. Examination of the cellular dynamics by IVM revealed that CD8+ T cells crawled along postcapillary venule endothelia at similar velocities during both ECM and hyperparasitemia (2.98±2.28 µm/min and 3.03±1.62 µm/min, respectively) (**Figure S3, Videos S6 and S7**). No extravascular CD8+ T cells were observed during hyperparasitemia. In agreement with previous reports, no differences were found in CD4+ T cells and densities between PbA and PyXL infections [26] (**Figure 3** **, Table S3, Video S8 and S9**). Together, these findings suggest that neurological signs and CD8+ T cell recruitment are correlated and occur rapidly.

#### Neutrophils

Significantly higher numbers of neutrophils were found in PCV and whole brain during ECM compared to HP. The density of GR-1 (Ly6G) positive neutrophils in postcapillary venules from PbA-infected mice with ECM on day 6–8 was 467.6±137.6 cells/mm^2^. No neutrophils were detected prior to ECM or in mice that survived the critical period without ECM development (**Figure 3A**, **Table S4**). PyXL-infected mice also exhibited neutrophils in postcapillary venules, albeit at the significantly lower density of 276.3±65.9 cells/mm^2^ at the time of hyperparasitemia (t-test: T_(5)_ = 2.80; *P*<0.05). Uninfected control mice did not exhibit arrested neutrophils. GR-1+ neutrophils did not extravasate into the perivascular space, neither during ECM nor during hyperparasitemia (**Videos S10 and S11**). Further, FTY720 treatment of PbA-infected mice reduced the number of neutrophils significantly so that levels similar to those found during HP were reached (**Figure 3C** **, Table S7**), which supports the previously suggested involvement of neutrophils in the ECM-associated vasculopathy and edema formation [26].

#### Monocytes and macrophages

Monocytes were present at similar densities in mice with ECM and HP (**Figure 3**, **Table S5**), which is consistent with earlier reports showing that monocytes are not involved in iRBC accumulation in the brain at the time of ECM [26]. The density of CD11b+ macrophages, however, was significantly higher during ECM (484.1±13.1 cells/mm^2^) compared to during hyperparasitemia (110.2±17.4 cells/mm^2^) (t-test: T_(2)_ = 16.56; *P*<0.01; **Figure 3A**, **Table S6**). In contrast to monocytes, which were exclusively found to be intravascular (**Figure 2** **, Videos S12 and S13**), CD11b+ macrophages were occasionally observed in the perivascular space (**Figure 2** **, Videos S14 and S15**). While macrophages could be tissue or blood derived, intravenous labeling suggests that the fluorescent extravascular cells are of the hematopoietic origin. However, we cannot exclude that the cells represent resident perivascular macrophages that were labeled by fluorescent marker leaking into the perivascular space.

#### Role of S1P receptors

We reported that the immunomodulatory drug FTY720 reduces the overall number of arrested leukocytes in the cortical microvasculature of PbA-infected mice [21], [39]. To determine the effect of FTY720 on CD8+ T cell arrest, mice were treated starting on day 1 prior to infection and on days 1, 3, and 5 after infection. FTY720 treatment allowed 73% of the mice to survive the critical period of ECM development (day 6–8) without exhibiting neurological signs until they were analyzed on day 9. Only ECM-negative mice were examined. While postcapillary venules from untreated mice contained 292.1±58.9 CD8+ T cells/mm^2^ at the time of neurological signs (**Table S2**), FTY720 treatment significantly reduced the cell density to 81.9±25.9 cells/mm^2^ (ANOVA; F_(2)_ = 59.41; *P*<0.001) (**Figure 3B** **, Table S2**). Despite the presence of these CD8+ T cells in cortical postcapillary venules, FTY720 treated mice failed to develop ECM. Interestingly, PyXL-infected mice with hyperparasitemia exhibited a similar density of CD8+ T cells, 61.0±8.1 cells/mm^2^ (**Table S2**), within postcapillary venules (Tukey's test: T = 1.59; ns). No CD8+ T cells were detected in the cortical microvasculature of uninfected control mice. Of note, neither the CD8+ T cells remaining after FTY720 treatment of PbA-infected mice nor those found in PyXL-infected mice with hyperparasitemia had any effect on the integrity of the BBB (**Figure 3B**). Thus, it appears that FTY720 treatment prevented a subpopulation of CD8+ T cells from traveling to the brain and that this coincided with the absence of vascular leakage and neurological signs.

### Leukocyte accumulation in the entire brain

To elucidate the composition of specific cellular subtypes involved in pathology we quantified leukocytes by flow cytometry in perfused whole brains. In contrast to IVM, no significant difference in CD8+ T cell recruitment was observed between ECM and HP suggesting that flow cytometry may lack the sensitivity to distinguish important focal variations in cellular composition as arrested leukocytes were not observed in other vessels such as capillaries or arterioles.

Overall, significantly more CD45+ leukocytes were found in the brains during ECM compared to hyperparasitemia (23729.3±7573.8 vs. 4483.0±2971.6; ANOVA: F_(2)_ = 12.42; *P*<0.001, Tukey's test: T = −4.49; *P*<0.05) and PbA/FTY720 mice (6059.7±4070.7; Tukey's test: T = −4.12; *P*<0.05) (**Figure 3C** **, Table S7**). Confirming the IVM data, ECM was associated with the recruitment of significantly higher numbers of Ly6G+ neutrophils than in hyperparasitemia (1470.7±325.5 vs. 518.0±317.2; ANOVA: F_(2)_ = 9.33; *P*<0.05), while there was no significant difference in the number of Ly6C+ monocytes.

The largest increase in cell numbers was observed for F4/80+ macrophages during ECM compared to hyperparasitemia (9648.0±3432.1 vs. 938.0±645.9; ANOVA: F_(2)_ = 14.35; *P*<0.01). Equivalent results were obtained for CD11b+ macrophages (ANOVA: F_(2)_ = 6.50; *P*<0.01). Because total brain leukocytes necessarily contain a large proportion of parenchymal macrophages, we distinguished these from blood-derived macrophages by their low level of CD45 expression [36], [56]. When the CD45^lo^ parenchymal macrophages (microglia) were excluded, the number of the remaining mostly intravascular CD45^hi^ F4/80+ macrophages was significantly higher during ECM compared to PyXL-infected mice (1750.0±285.0 vs. 243.3±171.5; ANOVA: F_(2)_ = 30.19; *P*<0.01) (**Figure 3D**).

FTY720 treatment of PbA-infected mice significantly reduced the number of Ly-6G+ neutrophils (Tukey's Test: T = −3.69; *P*<0.05), and both total and CD45^hi^ F4/80+ macrophages (Tukey's test: T = −4.31; *P*<0.05) in PbA-infected mice so that no significant difference was found for any of the cell types between PbA/FTY720 mice on day 9 and PyXL-infected mice with hyperparasitemia on day 5 (**Figure 3B-D** **, Table S7**). Equivalent results were obtained for CD11b+ macrophages (**Table S7**). Because neither FTY720-treated PbA-infected mice nor PyXL-infected mice with hyperparasitemia exhibit vascular leakage or neurological signs, it appears that FTY720 prevents BBB opening and the associated leukocyte recruitment, although we cannot exclude that FTY720 affects the brain directly.

### ECM-associated changes in leukocyte phenotype

**Figure 4:**
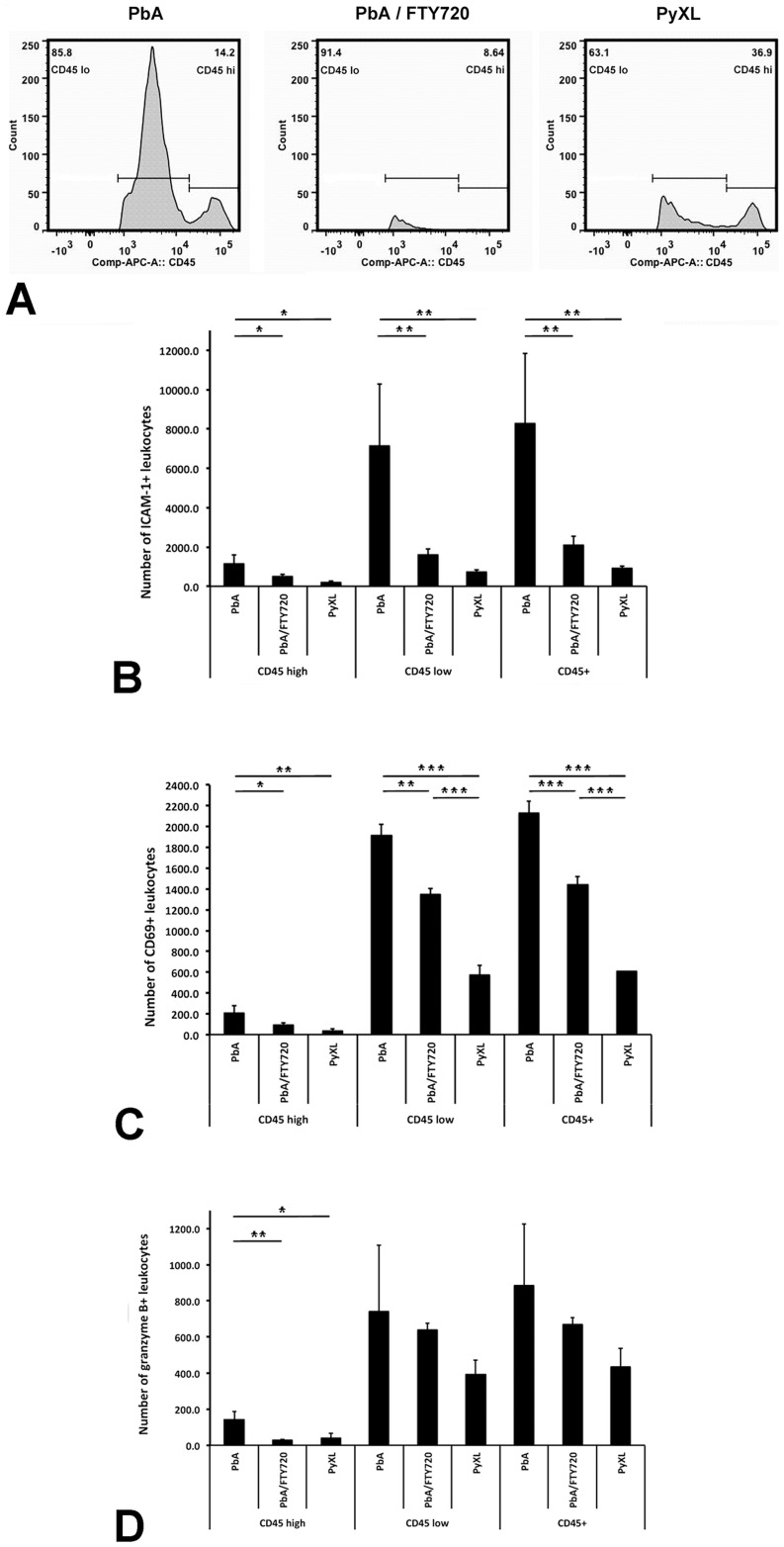
ECM is associated with brain recruitment of CD45+ leukocytes. **A**) PbA-infected mice exhibit significantly more CD45^hi^ and CD45^lo^ leukocytes in the brain compared to PyXL-infected mice. FTY720 treatment of PbA-infected mice reduces the number of CD45^hi^ and CD45^lo^ leukocytes to levels below those observed in PyXL-infected mice. ECM is associated with larger numbers of both CD45^hi^ and CD45^lo^ ICAM-1+ (**B**), CD69+ (**C**), and GrB+ (**D**) leukocytes compared to PyXL-infected mice. FTY720 treatment reduces the number of these leukocytes significantly, but not to the level found in PyXL-infected mice. Effect of FTY720 treatment reduces the number of GrB+ CD45^hi^ leukocytes. The data are based on groups of at least 3 mice per experimental condition. Significance (*, *P*<0.05; **, *P*<0.01) was determined with 1-way ANOVA followed by Tukey's test for multiple comparisons. See **Tables S8–S11** for details.

Flow cytometry revealed two distinct leukocyte subsets, namely CD45^hi^ and CD45^lo^ (**Figure 4A**), both of which were significantly more numerous in the brains of PbA-infected mice (ANOVA: F_(2)_ = 10.38; *P*<0.05 and ANOVA: F_(2)_ = 27.21; *P*<0.01, respectively) compared to PyXL-infected mice (Tukey's: T = −4.45; *P*<0.05 and Tukey's test: T = −7.37; *P*<0.01, respectively) (**Table S8**). FTY720 treatment significantly reduced the number of both CD45^hi^ (Tukey's test: T = −3.08; *P*<0.05) and CD45^lo^ (Tukey's test: T = −3.99; *P*<0.05) leukocytes. While the number of CD45^hi^ cells after FTY720 treatment was not statistically different from PyXL infection, the number of CD45^lo^ cells, although significantly decreased compared to PbA infection, remained significantly higher compared to PyXL-infected mice with hyperparasitemia (Tukey's test: T = −3.38; *P*<0.05) (**Table S8**). Significantly more ICAM-1+ (**Table S9**) and CD69+ leukocytes (**Table S10**) were present in the CD45^hi^ and the CD45^lo^ leukocyte subsets from PbA-infected mice compared to PyXL-infected mice (**Figure 4B–D**). FTY720 treatment significantly reduced the number of ICAM-1+, CD69+, and GrB+ leukocytes compared to PbA-infected mice with ECM (**Table S9, S10, and S11**). Further, the CD45^hi^ subset from PbA-infected mouse brains contained consistently higher numbers of ICAM-1+, CD69+, and GrB+ CD8+ T cells compared to PbA/FTY720 mice, although this difference was not statistically significant (**Figure S4A, Table S12**). Furthermore, the median expression levels of ICAM-1, CD69 and GrB in these CD45^hi^ CD8+ T cells were similar amongst PbA-infected, PbA/FTY720, and PyXL-infected mice (**Figure S4B-D**). Likely, the ECM-associated vasculopathy is caused by the high density of activated leukocytes in the postcapillary venules.

### Endothelial activation and expression of adhesion molecules

#### CD14 and PECAM-1 expression

Next, we explored molecular interactions that could potentially mediate leukocyte arrest to the microvascular endothelium. Various fluorescent markers were intravenously inoculated, which results in immunolabeling of molecules expressed on or bound to the luminal endothelial surface, while intracellular and extravascular binding sites remain undetected. Previous work indicated that postcapillary venules differ from similarly sized arterioles by differential expression of CD14 and CD31 [21]. PECAM-1 (CD31), generally considered a universal marker of vascular endothelia, was predominantly expressed in arterioles and somewhat less in capillaries. While PECAM-1 was constitutively low in postcapillary venules from infected and uninfected mice, independently of the experimental conditions, CD14 appeared at the time of ECM (day 6–8) on the endothelial surface of postcapillary venules, while capillaries and arterioles were negative [21]. Here we show that surprisingly, CD14 expression in postcapillary venules was not abrogated by FTY720 treatment of PbA-infected mice (**Figure S5, Videos S12 and S16**). In control mice, i.e. PyXL-infected mice with hyperparasitemia (day 5) and uninfected mice, the entire microvasculature including postcapillary venules was consistently CD14-negative (**Figure S5, Video S13**).

#### ICAM-1 expression on endothelia

**Figure 5:**
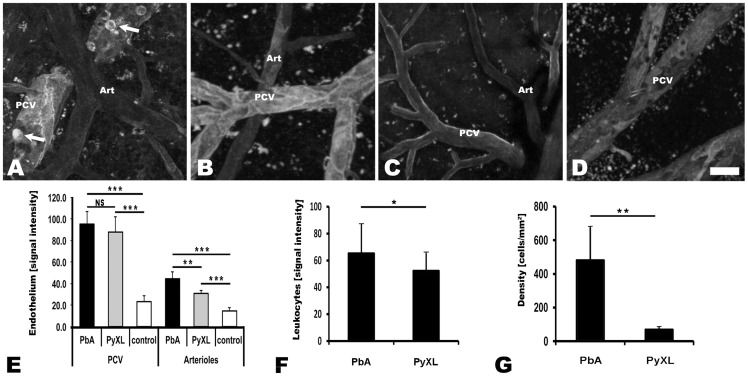
Both ECM and hyperparasitemia are associated with upregulation of endothelial ICAM-1. CBA/CaJ mice were infected with PbA, PyXL, or no parasites (control) and inoculated with PE-conjugated anti-ICAM-1 and Evans blue. **A–D**) Maximum projections of representative intravital microscopy movies showing endothelial ICAM-1 expression in postcapillary venules, and less so in arterioles (Art), in mice infected with PbA and PyXL compared to uninfected control mice. Note the pronounced ICAM-1 label along the endothelial junctions. **A**) PbA-infected mouse with ECM (day 6), **B**) PyXL-infected mouse with hyperparasitemia (day 5), **C**) uninfected control mouse. Note that ECM, but not hyperparasitemia, is associated with the expression of ICAM-1 on the surface of arrested leukocytes (light open circles, arrows). **D**) Endothelial ICAM-1 is upregulated, while ICAM-1 expressing leukocytes are absent, after FTY720 treatment of PbA-infected mice (day 9, no neurological signs). Scale bar = 20 µm. **E**) The endothelial ICAM-1 signal is similarly increased in cortical postcapillary venules from mice infected with PbA and PyXL compared to uninfected control mice. ICAM-1 is significantly upregulated in cortical arterioles during both ECM and hyperparasitemia compared to uninfected control mice. Compared to postcapillary venules, however, the overall level of ICAM-1 expression in arterioles is significantly lower. **F**) ICAM-1 fluorescence emission of individual leukocytes (N = 6) is higher during ECM compared to hyperparasitemia. **G**) The density of ICAM-1 expressing leukocytes in postcapillary venules from mice with ECM is significantly higher compared to mice with hyperparasitemia. Significance (*, *P*<0.05; **, *P*<0.01) was determined with 1-way ANOVA followed by Tukey's test for multiple comparisons. See **Table S13** for details and **Videos S17–S19** for the corresponding dynamic data.

Endothelial ICAM-1 plays a role in in leukocyte traversal across the BBB [57] and has been implicated in the pathogenesis of both HCM and ECM [58]–[62]. Intravenous labeling revealed that ICAM-1 was significantly upregulated (GLM: F_(3)_ = 154.14; *P*<0.001) in postcapillary venules from PbA-infected mice with ECM (**Figure 5A and 5E** **, Table S13, Video S17**) compared to uninfected control mice (Tukey's Test: T = 19.97; *P*<0.001) (**Figure 5C and 5E** **, Table S12**). Interestingly, ICAM-1 upregulation coincided with a labeling pattern that outlined the endothelial junctions. In agreement with earlier reports [63]–[65], ICAM-1 was also significantly upregulated in postcapillary venules from PyXL-infected mice with hyperparasitemia (**Figure 5B and 5E** **, Video S18**) compared to uninfected control mice (Tukey's Test: T = 18.79, *P*<0.001; **Figure 5C** **, Video S19**). No difference in endothelial ICAM-1 expression was found between PbA-infected mice with ECM and PyXL-infected mice with hyperparasitemia (Tukey's Test: T = −2.05; *P* = 0.324; **Table S13**), which is in agreement with the recruitment of leukocytes to postcapillary venules from both mice with ECM and hyperparasitemia [21]. Surprisingly, however, ICAM-1 was also significantly upregulated in arterioles, both in mice with ECM (Tukey's Test: T = 8.28; *P*<0.001) and hyperparasitemia (Tukey's Test: T = 4.73; *P*<0.001) compared to uninfected controls (**Figure 5E** **, Table S13**), although leukocyte arrest was not observed in this section of the microvascular tree. Further, ICAM-1 was also expressed in postcapillary venules from PbA-infected mice that had been treated with FTY720 (**Figure 5D**). Taken together, these findings do not support a role for endothelial ICAM-1 in ECM pathogenesis.

#### ICAM-1 expression on leukocytes

In contrast, IVM showed that ECM correlates with ICAM-1 upregulation on leukocytes (**Figure 5A**). The level of ICAM-1 expression per leukocyte was significantly higher (t-test: T_(10)_ = 2.58; *P*<0.05) during ECM (69.2±19.7; day 6–8, 6 mice) compared to hyperparasitemia (48.0±8.9; day 5, 6 mice) (**Figure 5F**). More strikingly, the density of ICAM-1 positive leukocytes in postcapillary venules was significantly higher (t-test: T_(8)_ = 4.30, *P*<0.01) during ECM compared to hyperparasitemia (**Figure 5G**). Thus, ECM is associated with the arrest of large numbers of ICAM-1+ leukocytes, consistent with a recent report on the contribution of leukocyte ICAM-1 to ECM development [66].

**Figure 6:**
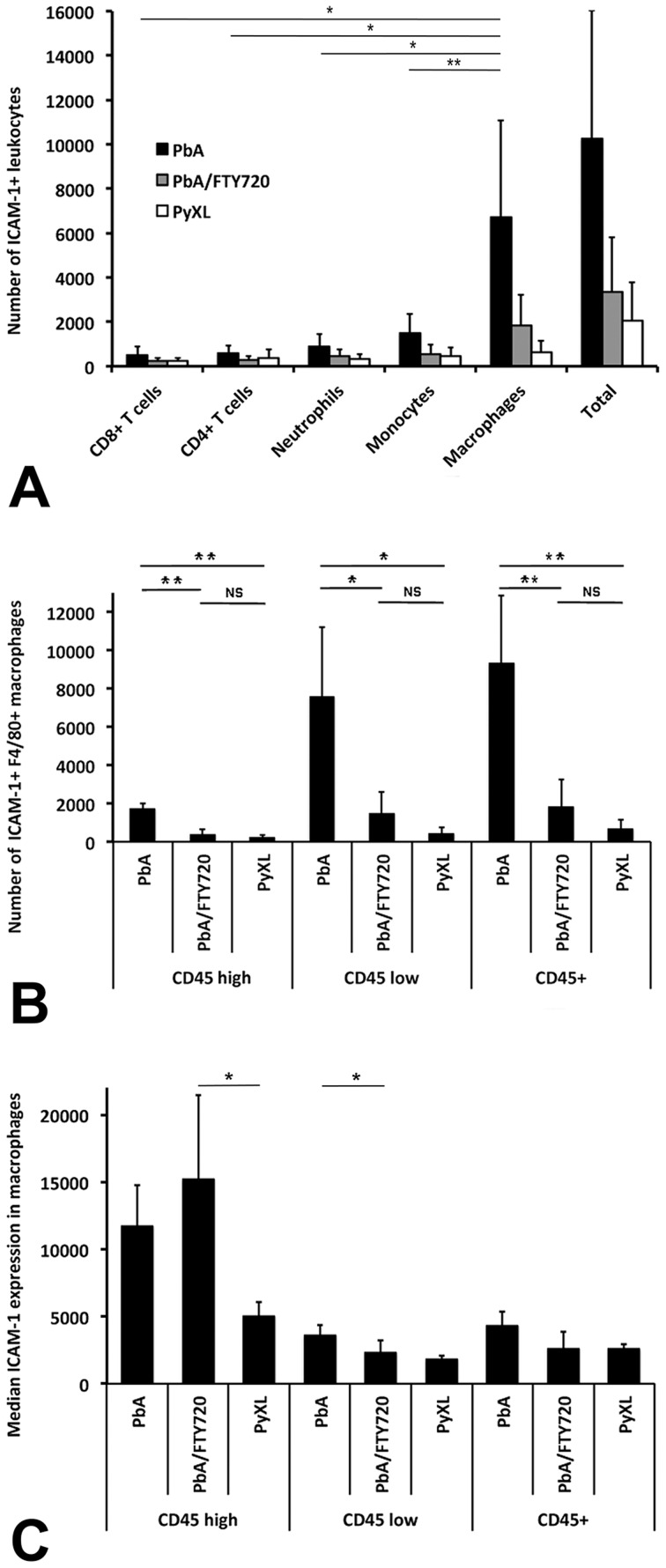
ECM is associated with the recruitment of ICAM-1+ macrophages. Leukocytes were isolated from PbA-infected, PbA-infected/FTY720-treated, or PyXL-infected mice and subjected to flow cytometric analysis. **A**) The vast majority of ICAM-1+ leukocytes are macrophages, which were identified with mAb F4/80. **B**) FTY720 treatment reduces the number of ICAM-1 macrophages to levels similar to those found in PyXL-infected mice. **C**) No significant difference was found for the median ICAM-1 expression levels in macrophages from PbA-infected versus PbA-infected/FTY720-treated mice. Significance (*, *P*<0.05; **, *P*<0.01) was determined with 1-way ANOVA followed by Tukey's test for multiple comparisons. See **Table S14 and S15** for details.

Flow cytometry identified the majority of these ICAM-1+ leukocytes as macrophages (**Figure 6A**). Macrophages constituted 65.5% and 54.5% of the ICAM-1+ leukocytes in the PbA and the PbA/FTY720 groups, respectively, but only 32.1% in the PyXL mice. Significantly higher numbers of ICAM-1+ CD45+ macrophages were present during ECM (ANOVA: F_(2)_ = 13.31; *P*<0.01) compared to PbA/FTY720 mice (9306.0±3552.2 vs. 1834.3±1414.9; Tukey's test: T = −4.76, *P*<0.01) (**Figure 6B** **, Table S14**). Interestingly, FTY720 treatment of PbA-infected mice reduced the number of ICAM-1+ F4/80+ macrophages to levels comparable to those found in PyXL-infected mice (656.7±504.1; Tukey's test: T = −0.65, *P* = 0.8; **Figure 6B** **, Table S14**). Similarly, there was a significant difference in the CD45^hi^ subset of ICAM-1+ F4/80+ macrophages (ANOVA: F_(2)_ = 30.10; *P*<0.01) between PbA-infected and PbA/FTY720 mice (1713.3±285.3 vs. 365.0±298.2; Tukey's test: T = −6.45; *P*<0.01; **Table S14**), i.e. the subset that is comprised mostly of blood-derived macrophages [36], [56]. FTY720 treatment of PbA-infected mice reduced the number of ICAM-1+ F4/80+ macrophages in the CD45^hi^ subset to levels similar to those found in PyXL-infected mice (219.0±161.6) so that again, similar numbers were found in the PbA/FTY720 and PyXL-infected groups (Tukey's test: T = −0.70, *P* = 0.773; **Table S14**). Equivalent results were obtained for CD11b+ macrophages (**Table S7**). Thus, ECM is associated with 1) the recruitment of large numbers of blood-derived ICAM-1+ macrophages to postcapillary venules and 2) a dramatic increase in the number of ICAM-1+ cells in the parenchyma of the brain, most likely microglia [36], [56]. As FTY720 treatment prevented this increase in ICAM-1+ macrophages, these data support the idea that the ECM-associated edema has both a vasogenic and a cytotoxic component [17], [36].

In agreement with the reported upregulation of ICAM-1 on macrophages under various other inflammatory conditions [67]–[70], we found higher, albeit statistically not significantly different, levels of ICAM-1 expression on F4/80+ macrophages in PbA-infected mice compared to PyXL-infected mice (4332.6±1007.0 vs. 2560.0±357.7; ANOVA: F_(2)_ = 4.47; *P* = 0.05) (**Figure 6C** **, Table S15**). Interestingly, FTY720 treatment of PbA-infected mice reduced the level of ICAM-1 expression in F4/80+ macrophages (2606.3±1258.1) resulting in levels similar to those found in PyXL-infected mice (2560.0±357.7). ICAM-1 expression on F4/80+ CD45^lo^ macrophages was significantly higher in ECM compared to hyperparasitemia (ANOVA: F_(2)_ = 5.57; *P*<0.05), but no difference in expression level was found between PbA/FTY720 with either ECM or hyperparasitemia infections (Tukey's test: T = −2.35.24, *P* = 0.122 and T = −0.79, *P* = 0.720, respectively). While ICAM-1 expression on F4/80+ CD45^hi^ macrophages was similar between ECM and hyperparasitemia, a significant difference was observed was between PbA/FTY720 and PyXL-infected mice (ANOVA: F_(2)_ = 5.40; *P*<0.05). Thus, ECM is associated with a greater number of ICAM-1+ macrophages (**Figure 6B**) and FTY720 treatment prevented this effect. It appears, therefore, that ICAM-1+ macrophages, not endothelia (**Figure 5**), are involved in the ECM-associated vasculopathy.

### ECM correlates with platelet arrest and P-selectin deposition in postcapillary venules

**Figure 7:**
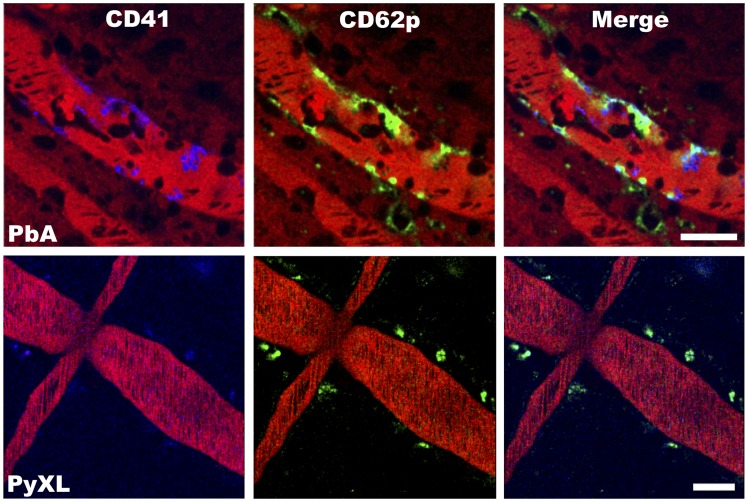
ECM correlates with the arrest of P-selectin expressing platelets in postcapillary venules. PbA-infected mice exhibit small clusters of CD41+ platelets (blue) and patches of P-selectin (green) along the wall of postcapillary venules at the time of ECM. Platelets remained in circulation and P-selectin was not detected in postcapillary venules from PyXL-infected mice with hyperparasitemia. The vascular lumen is visualized with Evans blue (bright red). Note that Evans blue has leaked into the brain parenchyma of the PbA-infected mouse (dark red shade on either side of the postcapillary venule). Scale bars = 50 µm. See **Video S20 and 22** for the corresponding dynamic data.

P-selectin release from platelet α-granules or endothelial Weibel-Palade bodies promotes the binding of platelets, leukocytes, and plasma proteins to the vascular wall [71]–[73]. Because both platelet marginalization and P-selectin expression have been implicated in the pathogenesis of both HCM and ECM [18], [20]–[22], [74]–[78], we determined the distribution of this adhesion molecule with respect to arrested platelets in the cortical microvasculature. Upon manifestation of neurological signs, PbA-infected CBA/CaJ mice were inoculated with a PE-conjugated mAb against P-selectin (CD62P), eFluor 450-conjugated anti-CD41 to detect platelets and Evans blue to visualize the vascular lumen [21]. IVM revealed small clusters of marginalized platelets that colocalized with patches of P-selectin on cortical postcapillary venule endothelia (**Figure 7** **, Video S20**). Occasionally, we observed strings of platelets that appeared to be attached to clusters of platelets (**Video S21**) as has been suggested to occur in HCM based on *in vitro* experiments [79]. In contrast, PyXL-infected mice with hyperparasitemia showed no evidence for P-selectin expression or platelet arrest (**Figure 7** **, Video S22**). Unlike postcapillary venules, arterioles were consistently negative for P-selectin or arrested platelets, both during ECM and hyperparasitemia. Thus, ECM, but not hyperparasitemia, is associated with marginalization of small numbers of platelets along postcapillary venule endothelia and P-selectin release, either from platelets or endothelia. However, the highly focal nature of both platelet arrest and P-selectin release contrasts with the uniform endothelial activation as evidenced by CD14 expression, ICAM-1 upregulation, and vascular leakage observed during ECM. Thus, leukocyte arrest is not limited to the P-selectin positive portions of the postcapillary venule endothelia.

### Endothelial junction protein expression is unaltered during ECM

FTY720 was previously shown to prevent vascular leakage, neurological signs, and death from ECM [21], [39]. To evaluate whether FTY720 protects the BBB by preserving the integrity of endothelial junctions [48], [80], [81], we determined the expression level of the tight junction (TJ) proteins claudin-5, occludin, and ZO-1 in the cerebral cortex and the cerebellum of 4 PbA-infected mice with ECM (day 6–8), 3 FTY720-treated PbA-infected mice that did not exhibit any neurological signs (day 8 or 9), and 3 PyXL-infected mice with hyperparasitemia (day 5) (**Figure S6 and S7**). Quantification of the fluorescence emission of specific antibodies on 3–4 immunolabeled cryostat sections per experimental condition yielded no significant reduction in protein expression under the different infection and treatment conditions compared to 3 uninfected control mice (**Table S16**). This finding suggests that the TJs remained morphologically intact and supports the hypothesis that the ECM-associated vascular leakage is based on a regulated, potentially reversible, mechanism of BBB opening [21], [82].

Thus, comparison of two *Plasmodium* infection models revealed: 1) The venous blood flow impairment during ECM is caused by the arrest of significantly higher numbers of CD8+ T cells, neutrophils, and in particular macrophages in cortical postcapillary venules compared to hyperparasitemia. While a small number of CD8+ T cells and macrophages extravasated into the perivascular space, most of the recruited leukocytes remained intravascular. 2) FTY720 treatment of PbA-infected mice reduced, but did not completely prevent leukocyte accumulation in postcapillary venules, which is consistent with the finding that low numbers of arrested leukocytes are present in PyXL-infected mice with hyperparasitemia without causing vascular leakage or neurological signs. 3) ECM closely correlates with the recruitment of large numbers of ICAM-1 expressing F4/80+ macrophages to the brain. As FTY720 treatment did not reduce the ICAM-1 expression level, the high density of these macrophages in postcapillary venules likely enhances the ECM-associated vascular pathology. 4) Leukocyte recruitment coincides with the onset of neurological signs, but follows BBB opening, as vascular leakage can be observed 1 day prior to symptomatic ECM [21].

## Discussion

**Figure 8:**
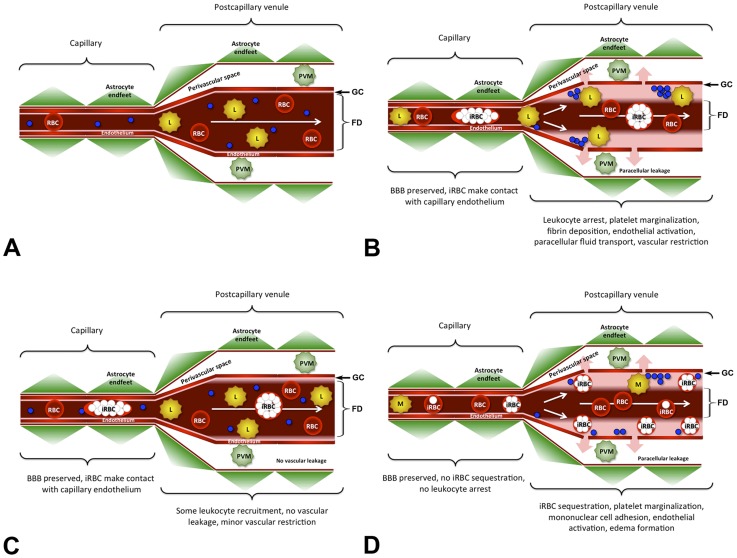
Model for the ECM-associated venous blood flow restriction. **A**) Under normal conditions, blood passes through the cerebral microvasculature without leukocytes arresting in postcapillary venules. Except for the narrow glycocalix (GC) lining the vascular endothelium, the entire vascular diameter is used for the bloodstream, the functional diameter (FD) is not restricted, and the BBB is intact. **B**) Infection with PbA causes endothelial junction opening at the BBB in the absence of junction protein degradation or endothelial death. During ECM, arrested leukocytes form steric obstacles that reduce the functional diameter (FD) of postcapillary venules resulting in a severe restriction of the venous blood flow. As a consequence, the paracellular leakage of plasma into the PVS (pink arrows) is exacerbated. Like uninfected RBC, iRBC travel with the bloodstream and do not arrest. **C**) During hyperparasitemia, significantly fewer leukocytes and no platelets adhere to the postcapillary venule endothelium compared to ECM. Consequently, the restriction in the venous blood flow is minor and there is no vascular leakage. **D**) Hypothetical model for pediatric *P. falciparum* HCM. While leukocytes, RBC, and all iRBC pass through capillaries without adhering to the endothelium, late-stage iRBC, mononuclear cells, and platelets sequester on the wall of postcapillary venules thus restricting the venous blood flow and exacerbating the leakage of plasma into the perivascular space. Because of the significant reduction in the functional diameter (FD) of the postcapillary venule, ring-stage iRBC, uninfected RBC, and most leukocytes must flow through the center of the vessel. The arterial blood flow remained unaffected under all experimental conditions. PVM = perivascular macrophage, iRBC = infected red blood cell, L = leukocyte, M = mononuclear cell, blue circles = platelets.

In this study, we identify a novel key determinant of ECM pathogenesis, namely that leukocyte arrest along the wall of postcapillary venules causes microrheological alterations that severely impair the venous blood flow. Based on our findings, we hypothesize that infection with PbA opens the BBB, which leads to the recruitment of numerous activated CD8+ T cells, ICAM-1+ macrophages, and neutrophils (**Figure 8**). The resulting steric hindrance of the blood flow in postcapillary and larger venules impairs, but does not block, the venous efflux from the brain, which exacerbates the vasogenic edema and causes death as a consequence of intracranial hypertension.

Under physiological conditions, the luminal surface of vascular endothelia is covered with a glycocalix, a 0.5 to >1 µm layer of membrane-bound proteoglycans and glycoproteins that repels RBCs and is critically involved in inflammatory responses, blood coagulation, and blood flow regulation [83]–[86]. IVM visualizes this glycocalix as a thin red layer, covering arteriolar endothelia from infected and uninfected mice and postcapillary venule endothelia from uninfected control mice. During ECM, the thickness of the RBC-free layer in postcapillary and larger venules was drastically increased. Because the glycocalix typically degrades under inflammatory conditions, leading to exposure of adhesion molecules, leukocyte adhesion, and impairment of endothelial barrier function [85], [86], the restriction in the venous blood flow during ECM is likely not caused by components of the glycocalix, but by increasing numbers of arrested leukocytes that prevent RBC from approaching the endothelium. Although the functional cross-section of postcapillary venules was occasionally reduced by more than 80%, complete vascular obstruction was not observed. These findings argue in favor of a combined vascular sequestration and immuno-pathological etiology of ECM [87]–[89].

The reduction in the venous blood flow must be expected to have major consequences for the physiology of the brain. First, the overall hypoperfusion of the brain, enhanced by inadequate contact between RBCs and the endothelium, likely contributes to the drastically reduced O_2_ delivery to the cerebral parenchyma observed in ECM-susceptible C57BL/6 mice [90]. In addition, by increasing the wall shear stress, leukocyte adhesion is expected to reduce the blood volume flow in postcapillary venules dramatically [91]. Finally, a reduction of the venous efflux from the skull, caused by a generalized narrowing of the lumen of venous microvessels, necessarily increases the intracranial pressure. The finding that brains from mice with ECM, but not hyperparasitemia, are swollen and spongy and bulge out of the skull, if the Dura mater is accidentally damaged during craniotomy clearly documents the dramatically increased intracranial pressure during the agonal phase of the disease. The reduced venous efflux from the brain may exacerbate vascular leakage, brain edema, and hemorrhages - cerebral alterations that are also associated with HCM [92]. Brain swelling and edema is extremely common in adult HCM on CT scan [93], [94]. Increased intracranial pressure has long been associated with poor prognosis and neurological sequelae in severe pediatric HCM [50], [95]–[98]. In fact, recent longitudinal MRI observations in Malawian children have identified intracranial hypertension as the single most important MRI finding associated with HCM development and the most reliable predictor of death [54], [99].

PbA-infected mice also exhibit arteriolar vasospasms during the final stage of ECM [100] and it has been suggested that the reduction in the cerebral blood flow observed by MRI [101] is due to increased production of vasoconstrictive factors or inhibition of vasodilating mediators [102]. Subsequent work [103], [104] revealed that endothelin-1 (ET-1), a potent vasoactive peptide with inflammatory and platelet-activating properties [105]–[108], is upregulated during both ECM and HCM [109]–[112]. Indeed, the arteriolar vasoconstrictive effect of ET-1 could be responsible for ECM induction in the PbA-infected C57BL/6 mouse model [108], because injection of exogenous ET-1 induces neurological signs in PbNK65-infected mice, which normally do not develop ECM [113], and because blockage of the ET-1 receptor A prevents ECM development in PbA-infected mice [110]. However, ET-1 has a plasma half-life of well under one minute in rodents [114] so that vasoconstriction alone cannot explain the increased intracranial pressure observed during ECM. Because ET-1 also stimulates endothelial activation with upregulation of adhesion molecules, promotes leukocyte adhesion, and increases vascular permeability [105], [106], there is a possibility that ET-1 induces ECM by restricting the venous blood flow. Similarly, administration of nitric oxide (NO), a key messenger involved in regulation of platelet adhesion and inflammatory and immune responses [115], decreased both leukocyte accumulation and vascular resistance in larger venules of PbA-infected mice [78], [116]–[121]. We conclude that the ultimate cause of death from ECM is a combination of arteriolar vasoconstriction and severe reduction in the blood efflux from the brain due to leukocyte adhesion in the venous microvasculature.

Compared to PyXL-infected mice with hyperparasitemia, PbA infection triggered the recruitment of significantly more CD8+ T cells to postcapillary venules at the time of, but not prior to, ECM development. Together with the finding that CD8+ T cells were absent in mice that survived the critical time for ECM development, these data suggest that neurological signs and T cell recruitment are correlated and occur rapidly. CD8+ T cells from C57BL/6 mice immunized with PyXNL can confer protection against lethal PyXL infection [122], whereas CD8+ T cell accumulation in the brain of PbA-infected C57BL/6 mice was abolished and the mice were completely protected from ECM when co-infected with *P. yoelii* [123], [124]. However, similar numbers of CD8+ T cells accumulated in the brains of PbA-infected C57BL/6 mice with ECM and PbNK65-infected mice without neurological signs [34], further supporting the notion that ECM-eliciting parasites such as PbA induce the recruitment of a qualitatively different CD8+ T cell population to the brain.

FTY720 treatment decreased the number of CD8+ T cells to levels similar to those found in PyXL-infected mice. Despite the presence of the remaining CD8+ T cells, neither FTY720-treated PbA-infected mice nor PyXL-infected mice developed neurological signs. A small percentage of CD8+ T cells entered the perivascular space during ECM, but not hyperparasitemia. This could be explained by upregulation of the leukocyte common antigen CD45, because CD45 expression is typically enhanced in response to stress signals, leading to increased leukocyte motility [125] and brain infiltration, for example after seizure [126]. ECM coincided with larger numbers of CD8+ T cells expressing CD69, one of the earliest lymphocyte activation markers [127], [128], and FTY720 treatment reduced the number of CD69+ CD8+ T cells to levels similar to those found during hyperparasitemia. Previous work supports a correlation between ECM and CD69+ CD8+ T cells: 1) Recruitment and activation of CD8+ T cells and CD69 expression were reduced in ECM-resistant mice [129]. 2) Peripheral CD8+ T cells were predominantly CD69+ during ECM and expressed the phenotype of memory T cells [130]. 3) The ECM-associated upregulation of CD69 was reversed and disease was prevented by interference with the angiotensin I pathway [131]. 4) Ghanaian *P. falciparum* infected pediatric patients with clinical HCM or severe anemia showed similar T cell activation profiles with a significantly increased frequency of CD69+ cells compared to asymptomatic children [132]. The median expression levels per cell of CD69 and GrB did not differ between the experimental groups suggesting that ECM pathogenesis correlates with a high density of CD69+ and GrB+ CD45^hi^ CD8+ T cells in postcapillary venules. FTY720 treatment of PbA-infected mice reduced the number of CD4+ T cells to levels similar to those observed for PyXL-infected mice. Together, these data suggest that FTY720 treatment prevents ECM by inhibiting the trafficking of activated CD8+ and CD4+ T cells to the brain.

FTY720 treatment of PbA-infected mice also reduced the number of macrophages and neutrophils to levels similar to those found in the brains of PyXL-infected mice, supporting the notion that these leukocytes exacerbate edema formation during ECM [26], [27]. Of particular importance, FTY720 treatment prevented the recruitment of large numbers of ICAM-1+ macrophages to the brains of PbA-infected mice. This finding sheds light on an unexpected new role of ICAM-1 in the pathogenesis of ECM. While FTY720 may preserve the integrity of the BBB primarily by preventing leukocyte recruitment to the brain, activated platelets release phosphorylated FTY720 [133], [134], which acts as a full agonist on the endothelial S1P receptor S1PR4; therefore, FTY720 may prevent the ECM-associated vascular leakage by strengthening the endothelial actin cytoskeleton [135], [136]. Further, FTY720 may regulate endothelial barrier function by directly modulating endothelial junction tightness, because the increased vascular leakage observed in mice deficient in plasma S1P can be reversed by restoring plasma S1P levels [47], [48], [80], [81]. This finding may explain why FTY720 administration must be started prior to the onset of vascular leakage [21], [39] and why attempts to rescue mice with symptomatic ECM, when leukocyte recruitment was already in progress, were unsuccessful (A. Movila and U. Frevert, unpublished observations). Further, FTY720 can cross the BBB and may thus be able to directly modulate parenchymal cells by interacting with S1P receptors in the CNS. The resulting feedback from the CNS on the activation status of the BBB may in turn alter the interaction with the immune cells. Future testing of FTY720 or related compounds in the ECM model is expected to reveal more detail on the exact mechanism of BBB opening and vascular inflammation in ECM. The ECM model may also improve our understanding of the pathogenesis of HCM. Ugandan, Malawian, and Central Indian children with HCM exhibit decreased S1P plasma levels compared to those with uncomplicated malaria and a low angiotensin-1 to angiotensin-2 plasma ratio discriminates HCM and severe non-cerebral from uncomplicated malaria and also predicts mortality from HCM [39], [137]–[140]. As these reports strongly suggest the involvement of the S1P pathway HCM, screening for novel immunomodulatory drugs and exploration of their endothelial barrier-promoting effects is warranted.

We observed significantly more neutrophils in postcapillary venules and whole brain during ECM compared to hyperparasitemia. Considering that the role of neutrophils in the pathogenesis of CM is understudied to date, this finding is of particular interest. FTY720 treatment of PbA-infected mice reduced the number of neutrophils significantly so that levels similar to those found during hyperparasitemia were reached, which supports the previously suggested involvement of neutrophils in the ECM-associated vasculopathy and edema formation [26], [141].

The number of arrested monocytes was increased similarly in PbA- and PyXL-infected mice compared to uninfected control mice suggesting that monocyte recruitment correlates with *Plasmodium* infection in general, not ECM in particular. This finding is in agreement with earlier reports showing that monocytes are not involved in iRBC accumulation in the brain at the time of ECM [26], [27]. Intravenous injection of fluorescent anti-CD14 revealed that monocytes were generally confined to the vascular lumen, although we occasionally detected labeled monocytes in the Virchow-Robin space. Similarly, intravenous injection of the macrophage marker anti-CD11b resulted in labeling of PVM. Because part of the PVM population derives from blood monocytes [53], these fluorescent monocytes may have been labeled before extravasating to replenish the pool of PVM in the perivascular space.

Flow cytometric measurement of leukocyte recruitment to the brain essentially corroborated our IVM findings suggesting that the cortical microvasculature reflects the ECM-associated pathological events in the entire brain. A few conceptual differences between IVM and flow cytometry are noteworthy. First, IVM confirmed the notion that the healthy murine brain has an extremely low level of immune surveillance with the almost complete absence of T cells, neutrophils, monocytes, and B cells [21], [142]. Expression of adhesion molecules is low on the healthy human postcapillary venule endothelium, and upregulation of P-selectin, E-selectin, ICAM-1, and VCAM-1 contributes to the pathogenesis of HCM [53], [58], [143]. Second, IVM provides information on the location of recruited leukocytes and their dynamics with respect to the complex and heterogeneous microvascular network of the brain. Third, intravenous labeling generally visualizes only intravascular leukocytes including those that extravasate between marker injection and imaging. However, the BBB at the level of postcapillary venules is naturally leaky, i.e. it allows passage of macromolecules including immunoglobulins into the Virchow-Robin space [38]. During ECM, this barrier becomes even more permeable, which makes distinction of recently extravasated from resident perivascular cells difficult. While CD8+ T cells do not patrol the cerebral microvasculature of uninfected mice and are therefore unlikely to extravasate into the perivascular space of naïve mice, the fluorescent CD11b+ macrophages we found in the perivascular space of mice with ECM could either have been labeled intravascularly and then extravasated or they could have been labeled after entering the perivascular space due to diffusion of the cell-specific markers across the BBB. Thus, while the majority of leukocytes remain confined to the vascular lumen during ECM, a small number of CD8+ T cells and possibly CD11b+ macrophages extravasate into the perivascular space. Fourth, flow cytometric measurement of the expression level of CD45 allowed distinction between blood-derived and parenchymal macrophages [56]. Using this approach, we found that ECM is associated not only with significantly higher numbers of ICAM-1+ CD45^hi^ ( = blood-derived) macrophages, but also ICAM-1+ CD45^lo^ ( = parenchymal) macrophages compared to mice with hyperparasitemia and FTY720-treated PbA-infected mice. Together with the ECM-associated activation of microglia [144] and the enhanced expression of CCR5 on non-hematopoietic cells [145], ICAM-1 upregulation on both blood-derived and parenchymal macrophages supports the notion of a mixed vasogenic and cytotoxic nature of the edema in ECM [17], [146]. Finally, IVM was critical to visualize endothelial activation markers within defined parts of the microvascular tree. Thus, IVM and flow cytometry provided highly complementary results.

BBB opening in the young CBA/CaJ mice used here begins at least one day prior to the onset of neurological signs, while leukocyte recruitment to the brain occurs only afterwards [21]. In 129,B6 mice, neither antibody-mediated neutrophil depletion nor clodronate-mediated macrophage depletion, done 1 or 2 days prior to ECM development, respectively, prevented neurological signs [26], [145]. While this was interpreted as neutrophils and macrophages not being involved in ECM development, an alternative explanation is that the BBB was already leaky at this late time point and the resulting vasogenic edema had already caused microglial activation. Clodronate liposomes do not cross the BBB [147]–[149] so that the microglia was likely unaffected and the resulting cytotoxic edema not prevented in these studies. Further, depletion of macrophages and granulocytes by administration of AP20187 to MAFIA mice on days 5, 6, and 7 after infection resulted in a >80% reduction of these cells in the blood [27], [150]. Again, as AP20187 does not cross the BBB [151], ECM development likely manifested because the activated microglia was not eliminated. Importantly, CD8+ T cells alone failed to induce any of the typical signs of ECM including convulsions and death without the direct neurotoxic effect of intravenously administered folic acid [26], [145]. Thus, it appears that both hematogenic and parenchymal macrophages play a role of in the pathogenesis of ECM. A more recent study in C57BL/6 mice emphasizes the crucial role of monocytes/macrophages in lymphocyte recruitment to the brain. Clodronate treatment 2 days prior to manifestation of neurological signs reduced the recruitment of CD8+ T cells, CD4+ T cells, and NK cells to the brain 2.8-fold, 1.8-fold, and 4.6-fold, respectively, and failed to alter the course of disease development [152]. Clodronate treatment 2 days prior to infection with PbA, on the other hand, prevented ECM development [152]. Thus, the exact mechanism by which blood-derived macrophages contribute to disease development needs further and more specific investigation. Taken together, these data suggest the following scenario: PbA infection induces BBB opening, which results in vasogenic edema and microglial activation. The ensuing cytotoxic edema then leads to endothelial activation with recruitment of leukocytes that in concert restrict the venous blood flow, which further weakens the already impaired BBB. Eventually, these events culminate in the typical irreversible damage associated with fatal malarial encephalopathy. However, the differential progression of the vasogenic versus the cytotoxic edema and their relative contribution to brainstem compression and death clearly require further experimental attention.

The critical role of ICAM-1 in the pathogenesis of severe *P. falciparum* malaria and the binding of *P. falciparum* iRBC to the endothelium is generally accepted [59], [61], [153]–[159]. However, comparison of the endothelial ICAM-1 expression levels in the brains of *P. falciparum* versus *P. vivax* infected individuals is necessary to determine whether or not HCM correlates with upregulation of ICAM-1 in postcapillary venules. ICAM-1 upregulation has also been implicated in the pathogenesis of ECM [63], [77], [78], [160]–[162]. While the cellular origin of ICAM-1 was not determined, treatment of PbA-infected mice with NO reduced ICAM-1 upregulation in the brain along with leukocyte sequestration and vascular leakage [78]. We show that upregulation of endothelial ICAM-1 correlates with *Plasmodium* infection in general, not with ECM in particular. Because ICAM-1 upregulation was not observed in mice deficient in RAG-1 or IFN-γ on day 6 after infection with PbA [163], the Th1 type cytokines IFN-γ and lymphotoxin, in synergy with TNF from macrophages [163], [164], may induce ICAM-1 expression throughout the brain microvasculature of ECM-susceptible mice, both during ECM and hyperparasitemia. Together with the finding that FTY720 treatment of PbA-infected mice does not prevent ICAM-1 upregulation in postcapillary venules, these data argue against a role of this endothelial adhesion molecule in the pathogenesis of ECM.

CD14 appeared on the surface of postcapillary venule endothelia selectively at the time of neurological signs (day 6–8), which is in agreement with the well-known involvement of CD14 in endothelial activation, neuroinflammation, and leukocyte recruitment to the cerebral microvasculature [165]–[168] and the finding that CD14-deficient mice are protected against ECM [169]. Further, because CD14 plays an important role in the non-phlogistic clearance of apoptotic cells [170], its expression on postcapillary venule endothelia may reflect the well-documented increase in apoptosis during ECM [171]–[173]. Interestingly, malaria-associated microparticles carry phosphatidylserine on their surface [174]. Because the plasma levels of endothelial, platelet, and erythrocytic microparticles increase at the onset of the ECM-associated neurological signs [175], CD14 may be involved in the recruitment of microparticles to postcapillary venule endothelia. Platelet-derived microparticles are known to promote macrophage differentiation [176] and may contribute to the observed upregulation of ICAM-1 on the macrophage surface.

We show that ECM occurs without apparent physical degradation of endothelial TJs, measurable loss of claudin-5, occludin, and ZO-1 and in the absence of endothelial death [21]. Together, these findings argue against widespread and irreversible endothelial injury, for example due to cytotoxic T cell-mediated apoptosis or long-lasting vascular occlusion [177], as a major pathogenetic mechanism leading to malarial coma and death [82], [88], [178], [179]. Instead, and in agreement with the observation that ECM-susceptible mice can be rescued by anti-LFA-1 treatment even minutes before imminent death [180], [181], the preserved TJ integrity suggests that BBB opening during ECM is under the control of a regulated mechanism [21], [82], [182]. In support of this notion, fast-acting anti-malarial drugs can prevent ECM one day before the expected onset of neurological signs, although CD8+ T cells still accumulate in the brain [32], [34].

Platelets play a crucial role in the early stages of the pathogenetic cascade of ECM [9], [183], [184]. Blockage of platelet activation or shedding of platelet-derived microparticles confers resistance to ECM [13], [22], [160], [184]. At the time of ECM, platelets arrest focally and in small numbers in the cortical microvasculature [21] where they closely associate with P-selectin positive areas on postcapillary venule endothelia, confirming the reported involvement of both platelets [18], [20]–[22], [74]–[76] and P-selectin [77], [78] in the pathogenesis of HCM and ECM. Although platelets produce many factors including VEGF, ROS, and thrombin that are able to directly impair endothelial barrier function [185], the highly focal nature of platelet arrest [20], [21] is difficult to reconcile with the homogenous vascular leakage throughout the entire venous microvasculature at the time of ECM [21]. In agreement with their crucial role early in the pathogenesis of ECM, platelets are more likely to accomplish their vascular permeability-augmenting effect indirectly, namely by 1) facilitating leukocyte arrest in postcapillary venules [22], [57], [186], [187], 2) boosting ROS formation in leukocytes [185], and 3) enhancing cytokine secretion and cytotoxic capacity of effector T cells [188]. Thus, platelets adhering to small foci of endothelial P-selectin may serve as early nucleation points for the gradually spreading, cytokine-induced vasculopathy observed in postcapillary venules from ECM-susceptible mice.

In conclusion, we find that steric hindrance, mediated by large numbers of arrested CD8+ T cells, macrophages, and neutrophils in postcapillary venules, causes a severe restriction in the venous blood flow. Based on this observation, we hypothesize that completely different pathogenetic mechanisms - cytoadherence of *P. falciparum* iRBC in HCM and sequestration of leukocytes in ECM - can result in the same pathophysiological outcome: a severe reduction in the blood efflux from the brain. If the resulting increase in intracranial pressure intensifies the cerebral edema to the point of brainstem herniation, then compression of the respiration centers in pons and medulla could cause death by respiratory arrest.

Intracranial hypertension is a known risk factor for poor outcome in pediatric HCM [189], [190], but how this leads to neural injury is unknown [191], [192]. As discussed in a recent review [193], intracranial hypertension eliminates the need to explain any selective recognition mechanism *Plasmodium* might use to target multiple sensitive sites in the brain [194]. Intracranial hypertension leading to general swelling and hypoperfusion of the brain can also explain all of the neurological sequelae in HCM survivors [194]. Reports associating loss of smell, deafness, and blindness with both HCM and ECM support the notion that sequestration of iRBC as well as leukocytes can cause intracranial hypertension [192], [195]–[200]. Further, leukocyte sequestration is likely also involved in intracranial hypertension during *P. falciparum* HCM, as artesunate treatment was more efficacious in Asian adults compared to African children with more mononuclear cell accumulation [157], [201], [202] and also failed to rescue HCM patients with a low parasite biomass in the brain [203]. Thus, HCM and ECM induce very similar neurological symptoms and sequelae.

Thus, despite fundamental differences in parasite biology, the non-cytoadherent rodent parasite PbA could be used as a model to better understand how rheological alterations might lead to the annual death from HCM of over half a million people [204]. Perhaps more importantly, venous efflux disturbances due to leukocyte sequestration could also explain the cerebral complications that are increasingly reported for severe infections with *P. vivax*, another knobless (essentially non-cytoadherent) *Plasmodium* species [205]–[214]. The marked proinflammatory responses and reversible microvascular dysfunction associated with *P. vivax* infections [215], [216], together with the shared propensity for reticulocyte invasion [217]–[220], may render the PbA-infected mouse model suitable for study of the pathogenesis of severe *P. vivax* malaria. Hence, this model promises to shed light on the ultimate cause(s) of death from cerebral malaria.

## Materials and Methods

### Ethics statement

This study was conducted in strict accordance with the recommendations in the Guide for the Care and Use of Laboratory Animals of the National Institutes of Health. The protocol was approved by the Institutional Animal Care and Use Committee, NYU School of Medicine (Protocol number 101201-01). All surgery was performed under ketamine-xylazine-acepromazine anesthesia, and all efforts were made to minimize suffering.

### Parasites

*P. berghei* and *P. yoelii* parasites were maintained by passage through female *Anopheles stephensi* mosquitoes [221]. The green fluorescent *P. berghei* ANKA strain (PbA-GFP) was a kind gift from Dr. Andy Waters, University of Glasgow, UK [222]. The lethal *P. yoelii* strain 17XL, originally derived from the non-lethal 17X strain [223], was kindly provided by Dr. James Burns (Drexel University College of Medicine) [224], [225]. WT PyXL were transfected to express RedStar, an improved version of the red fluorescent protein drFP583/DsRed/RFP [226], under the control of the elongation factor 1α promoter using a novel replacement strategy [21], [227], [228] and termed PyXL-RFP [21]. Both PbA-GFP and PyXL-RFP emit fluorescence throughout the entire life cycle.

### Mice and infection

Mice were CBA/CaJ (Jackson Laboratory, Bar Harbor, ME). Animals were maintained and used in accordance with recommendations in the guide for the Care and Use of Laboratory Animals. CBA/CaJ mice were infected at the age of 3 weeks (body weight of 12–15 g) by intraperitoneal injection of 0.5–10×10^6^ iRBC as described [21]. In our hands, 3 week-old CBA/CaJ mice responded to PbA infection with neurological signs and died from ECM comparable to adult mice used by others [3], [6], [18], [22], [23], [76], [141], [169], [229]–[244]. The parasitemia was monitored daily using Giemsa stained blood smears and mice were sacrificed upon development of ECM or hyperparasitemia.

### Assessment of neurological signs

PbA-infected mice were considered ECM positive when two or more parameters clearly indicated behavioral alteration including body position, spontaneous activity, startle response, tremor, gait, touch escape, and righting reflex [245]. Quantitative assessment of ECM-associated neurological signs was performed using the Rapid Murine Coma and Behavior Scale (RMCBS) with values of 3–7 defined here as severe, 8–16 as mild, and 17–20 as no ECM [246]. PyXL-*infected* mice exhibited a hunched position, pale skin color, and an increased respiration rate when parasitemia levels reached levels around 80%, but failed to present typical neurological manifestations.

### FTY720 treatment

For IVM, groups of 15 PbA-infected CBA/CaJ mice received one daily oral dose of 0.3 mg/kg FTY720 starting one day before infection or no treatment as described [21], [39]. At the onset of neurological signs, mice were injected with Evans blue and examined by IVM. Mice surviving the critical period of ECM development were inoculated with Evans blue and PE-conjugated species anti-species CD8a and imaged on day 9. Groups of 5 PyXL-infected or uninfected mice were inoculated with the same markers and imaged on day 5 for comparison. For flow cytometry, PbA-infected mice, FTY720-treated PbA-infected mice, and PyXL-infected mice were analyzed on day 6–8, day 8, and day 5, respectively.

### Anesthesia, craniotomy, and intravital microscopy

Mice were anesthetized by intraperitoneal injection of a cocktail of 50 mg/kg ketamine (Ketaset, Fort Dodge Animal Health, Fort Dodge, IO), 10 mg/kg xylazine (Rompun, Bayer, Shawnee Mission, KS), and 1.7 mg/kg acepromazine (Boehringer Ingelheim Vetmedica, St. Joseph, MO) (KXA mix) and surgically prepared for intravital imaging of the brain as described [21], [247], [248]. CBA/CaJ mice were infected with PbA and subjected to brain IVM 1) on day 5 prior to the appearance of neurological signs, 2) on day 6–8 upon ECM development, or 3) on day 9 after the window of ECM development had passed. PyXL-infected mice were imaged upon the parasitemia exceeding 50% on day 5. Uninfected mice were used as controls. Prior to imaging, mice were inoculated with Evans blue and matching combinations of fluorescent markers. Despite severe illness of the animals, optimization of anesthesia, craniotomy, and injection of fluorescent markers allowed us to obtain good recordings from approximately 70% of the mice with ECM and 50% of the mice with hyperparasitemia.

### Monitoring cell density and dynamics in the cortical microvasculature

iRBC were identified by fluorescent protein expression in the parasites or reflection of hemozoin [21]. The vascular lumen was visualized by intravenous injection of 100 µl of a 1% solution of Evans blue. Vascular endothelia were labeled intravenously with Alexa 488 or eFluor 450-conjugated rat anti-mouse PECAM-1 (CD31; clone MEC13.3, BioLegend, San Diego, CA) and phycoerythrin (PE)- or Alexa 647-conjugated rat anti-mouse CD14 (clone Sa2-8, eBioscience). CD8+ T cells, CD4+ T cells, monocytes, macrophages, neutrophils, or platelets were labeled by intravenous injection of 3–5 µg of the following fluorochrome-conjugated monoclonal antibodies using appropriate color-matching combinations: PE-conjugated rat anti-mouse CD8a (clone 53-6.7; eBioscience, San Diego, CA), eFluor 450 or PE-conjugated rat anti-mouse CD4+ (clone GK 1.5, eBioscience), Pacific blue-conjugated rat anti-mouse CD11b (clone M1/70, BioLegend), eFluor 450-, PE- or Alexa 647-conjugated rat anti-mouse GR-1 (Ly-6G/6c; clone RB6-8C5, eBioscience and BioLegend), and eFluor 450-conjugated rat anti-mouse CD41 (clone MWReg30, eBioscience), respectively. ICAM-1 expression was visualized with intravenously inoculated PE-conjugated rat anti-mouse CD54 (clone YN 1/1.7.4, Biolegend). P-selectin was detected with PE-conjugated rat anti-human/mouse CD62p (KO2.3, eBioscience).

### Quantification of leukocyte arrest and ICAM-1 expression

Multiple time sequences and 3D stacks were recorded for quantification of the number of arrested leukocytes in the vascular lumen. Depending on the experimental conditions, 20–45 postcapillary venules and arterioles were analyzed per mouse. Arrested leukocytes were defined in each vessel segment as cells that did not detach from the endothelial lining within the observation period. CD8+ T cells, CD4+ T cells, neutrophils, monocytes, and macrophages were quantified by counting the number of cells per square millimeter of vessel surface [249]. The relative density of the various leukocyte populations was determined in multiple fields of view per experimental condition and expressed as the mean ± SEM of arrested cells as well as the percentage of the total cell number. Velocities were measured with Imaris Track as described [250].

To quantify endothelial ICAM-1 expression, confocal 3D stacks of postcapillary venules or similarly sized arterioles were acquired from 2 mice each infected with PbA, PyXL, or no parasites. The fluorescence signal intensity across 10 (PbA) or 12 (PyXL and control) vessel volumes was collected from 3D data and quantified in ImageJ. To quantify leukocyte ICAM-1 expression, the relative fluorescence emission from individual leukocytes was measured from mice infected with PbA or PyXL. As for endothelial ICAM-1 expression, confocal 3D stacks were collected and projections were created in AutoDeblur. A total of 6 stacks from 2 mice per experimental condition were analyzed.

### Brain leukocyte harvesting and flow cytometry

Leukocytes were prepared from the brain (cerebrum and cerebellum) using established procedures [251]–[253]. Briefly, mice were perfused intracardially with Mg^2+^ and Ca^2+^-free PBS to dislodge nonadherent leukocytes. Next, the brain was removed and gently minced through a mesh strainer (mesh size: 100 µm; Fisher Scientific) using a syringe plunger. The homogenate was suspended in 10 ml HBSS containing 0.05% collagenase D (Roche Diagnostics, Indianapolis, IN), 0.1 µg/ml of the trypsin inhibitor TLCK (Sigma), 10 µg/ml DNase I (Sigma), and 10 mM Hepes buffer, pH 7.4. The tissue slurry was gently rocked at RT for 60 min and then allowed to settle at 1 g for 30 min. The supernatant was collected and 5 ml of the suspension was layered onto 10 ml of a density separation medium containing 7.5 ml of RPMI medium containing 10% FBS, 10 mM HEPES, and 2.5 ml Ficoll Paque (GE HealthCare) in a 50 ml conical centrifuge tube and centrifuged at 400 g for 30 min. The overlying media and tissue debris were removed, the entire gradient medium was diluted ten-fold with HBSS and centrifuged at 300 g for 10 min. Isolated leukocytes were washed twice with Mg^2+^ and Ca^2+^-free PBS before phenotyping. A total of 10 PbA-infected mice with ECM, 6 PbA-infected/FTY720-treated, and 6 PyXL-infected mice with hyperparasitemia were subjected to flow cytometric analysis. Uninfected control mice were not included in the analysis, because the cerebral microvasculature of these animals does not exhibit arrested leukocytes.

The following antibodies were used for leukocyte phenotyping: Total leukocytes were detected with PE-Cy7-conjugated rat anti-mouse CD45 (clone 30-F11; eBioscience, San Diego, CA), T cells with APC-Cy7-conjugated Armenian hamster anti-mouse CD3 (clone 145-2C11; BD Biosciences, San Jose, CA), CD8+ T cells with eFluor 450 or Alexa 700-conjugated rat anti-mouse CD8a (clone 53-6.7; eBioscience or Biolegend, San Diego, CA), CD4+ T cells with PE- Texas Red-conjugated rat anti-mouse CD4+ (clone RM4-5; Life Technologies, Carlsbad, CA, neutrophils with FITC-conjugated rat anti-mouse Ly6G (clone 1A8; Biolegend), monocytes with Alexa 700-conjugated rat anti-mouse Ly6C (clone HK 1.4; Biolegend), and macrophages with anti-mouse CD11b (clone M1/70; eBioscience) or PE- or eFluor 450-conjugated anti-mouse F4/80 (clone BM8; eBioscience). CCR5 (CD195) was detected with PE-conjugated rat anti-mouse CD195 (clone HM-CCR5; Biolegend), CD69 with Pacific Blue-conjugated rat anti-mouse CD69 (clone H1.2F3; Biolegend), ICAM-1 with APC-conjugated rat anti-mouse CD54 (clone YN1/1.7.4; eBioscience), and granzyme B with FITC-conjugated anti-mouse granzyme B (clone GB11; Biolegend). Data were acquired with a 5-laser, 17-color LSR II Analytic Flow Cytometer (BD Biosciences, San Jose, CA) and analyzed with FlowJo software (Treestar, Ashland, OR).

### Microrheology

Time series showing the blood flow in postcapillary venules or similarly sized arterioles were acquired from mice infected with PbA, PyXL, or no parasites and converted to minimal projections to visualize the portion of the vascular lumen used for blood flow. The vascular lumen was visualized by IV inoculation of Evans blue [21]. IVM movies were converted to minimal projections to visualize the perfused center of the vessel. Multiple measurements were taken for each vessel to determine the entire vascular diameter (distance between endothelia) versus the perfused part of the vessel (dark center) (**Figures 1** **and S2**). Vascular restriction is expressed as percent reduction in vessel diameter or cross-section.

### Intravital confocal microscopy and image processing

The cortical microvasculature was imaged with an inverted Leica TCS SP2 AOBS confocal system as described [21]. Time series and 3D data sets were acquired with Leica Confocal Software [248], [250], [254]. Imaris 7.4 (Bitplane, Saint Paul, MN), Image-Pro Plus (Media Cybernetics, Bethesda, MD), AutoDeBlur (Media Cybernetics, Bethesda, MD), and NIH ImageJ were used for further image analysis, deconvolution, and 3D reconstruction.

### Endothelial junction integrity

Blood was removed by perfusion with PBS via the left ventricle [251]. Brains were snap-frozen for preparation of cryostat sections. To determine the expression level of tight junction proteins, sections were fixed in 95% ice-cold ethanol for 30 minutes and then permeabilized in acetone for 1 min at RT. After blocking in 50% goat serum for 45 min, sections were labeled with rabbit polyclonal antibody against claudin 5 (34-1600), rabbit polyclonal anti-occludin (40-4700), mouse monoclonal anti-ZO1 (33-9100), all from Invitrogen. Rat monoclonal antibody anti-CD31 (553370) was from BD Biosciences. Sections were then incubated for 2 h at RT with secondary Alexa antibodies (Life Technologies) and mounted in Vectashield with DAPI (H1200, Vector Labs, Burlingame, CA). Depending on the experimental condition, 9–16 images from various regions of the cerebrum and cerebellum were analyzed. Data were acquired at the same magnification with Zeiss Axiovison software using an Axio Imager-D2 Zeiss fluorescent microscope and imported into Image J for quantification of vascular leakage and tight junction protein expression. For tight junction protein expression, the fluorescence threshold was set with the “triangle” setting of Image J to limit the measurement of the brightest signal only, corresponding to the tight junctions. Ratios were then calculated by comparing the experimental groups with the control group using Excel. Significance was determined by t-test.

### Statistical analysis

Statistical analysis was performed using Minitab® 17 (Minitab, State College, PA). Data sets were tested for normality and equal variances and when necessary data was Log10 transformed to obtain a normal distribution. Data were analyzed either by t-test, one-way ANOVA, or General Linear Model as appropriate. Where transformation was not successful, the non-parametric Mann-Whitney U test was used.

## Supporting Information

Figure S1 **Confocal intravital microscopy of the cortical microvasculature.** Intravital microscopy of a PbA-infected CX3CR1^GFP/+^ mouse with ECM shows that the postcapillary venules, capillaries, and arterioles analyzed in this study are embedded in green fluorescent microglia (arrows) and thus located in the cerebral cortex, i.e. below the layer of pial microvessels. Evans blue (red) visualizes the microvascular lumen, while blood cells are negatively stained (black). Leukocytes crawl slowly (large black circles), while RBCs move at bloodstream velocity and appear as dark streaks. Note that the vascular marker has leaked into the perivascular space. Scale bar = 50 µm.
(TIF)

Figure S2 **Visualization of the luminal restriction of postcapillary venules during ECM.** Examples of intravital movies and minimal projections of postcapillary venules from PbA-infected mice with ECM. **A, C**) Individual frames show postcapillary venules containing blood cells (dark circles or streaks) that are distorted due to the relatively slow confocal scan speed compared to the much faster blood flow. **B, D**) Minimal projections of the movies reveal that the functional vessel diameter (short arrows), i.e. the perfused portion of the vessel, is considerably reduced compared to the entire vessel diameter (long arrows). Visualization of the vascular lumen with Evans blue reveals a red zone along the endothelium of postcapillary venules that is devoid of RBC (dark). Scale bars = 20 µm. See **Video S1 and 2** for the corresponding dynamic data.
(TIF)

Figure S3 **CD8+ T cell velocity during ECM and hyperparasitemia.** CBA/CaJ mice infected with PbA or PyXL were inoculated with PE or eFluor 450-conjugated anti-CD8+a at the time of ECM or hyperparasitemia, respectively, and velocity and density of CD8+ T cells was determined by off-line analysis of intravital microscopy time sequences and 3D stacks, respectively. Mean velocity of intravascular CD8+ T cells during ECM (PbA) and hyperparasitemia (PyXL). The data represent the mean ± SEM of 61 cells from 5 PbA-infected and 10 cells from 5 PyXL-infected mice. Significance was calculated with 1-way ANOVA.
(TIF)

Figure S4 **ICAM-1, CD69, and GrB expression in CD8+ T cells.** Leukocytes were isolated from the brains of PbA-infected, PbA-infected/FTY720-treated, and PyXL-infected mice. **A**) Flow cytometry reveals that FTY720 treatment reduces the ECM-associated accumulation of ICAM-1+ CD69+ GrB+ CD45^hi^ CD8+ T cells in the brain of PbA-infected mice to levels similar to those found in PyXL-infected mice with hyperparasitemia. The data are based on groups of at least 3 mice per experimental condition. Significance was determined with 1-way ANOVA followed by Tukey's test for multiple comparisons. See **Table S12** for details. **B–D**) Flow cytometry revealed no significant difference in the median expression levels of ICAM-1 (CD54) (**B**), CD69 (**C**), or GrB (**D**) in the CD45^hi^ subset of CD8+ T cells compared to PyXL-infected or PbA-infected/FTY720-treated mice. Data are based on 3 mice per group. Significance (*, *P*<0.05) was determined with 1-way ANOVA followed by Tukey's test for multiple comparisons.
(TIF)

Figure S5 **Effect of FTY720 on endothelial CD14 expression.** Postcapillary venule endothelia of PbA-infected mice are positive for CD14 at the time of ECM (green outline) (**A**). FTY720 treatment does not prevent endothelial CD14 expression in postcapillary venules (green outline) (**B**). PyXL-infected mice do not exhibit endothelial CD14 at the time of hyperparasitemia (**C**). Mice were inoculated with Evans blue (red) to visualize the vascular lumen and PE-conjugated anti-CD14. Note that monocytes (green open circles) are also CD14 positive (arrows in A and C). See **Videos S12, S13, and S16** for the corresponding dynamic data.
(TIF)

Figure S6 **TJ protein expression in the cerebral cortex.** Cryostat sections of the brains of PbA-infected CBA/CaJ mice with ECM (day 6–8; N = 4), PbA-infected and FTY720-treated mice that did not exhibit any neurological signs (day 8 or 9; N = 3), and PyXL-infected mice with hyperparasitemia (day 5; N = 3) were immunolabeled with specific antibodies the TJ proteins claudin-5, occludin, and ZO-1. Multiple confocal microscopy images per experimental condition were acquired and the overall TJ protein-specific fluorescence emission was quantified with ImageJ. No significant reduction in TJ protein expression was detected under the different infection and treatment conditions compared to uninfected control mice (N = 3).
(TIF)

Figure S7 **TJ protein expression in the cerebellum.** Cryostat sections of the brains of PbA-infected CBA/CaJ mice with ECM (day 6–8; N = 4), PbA-infected and FTY720-treated mice that did not exhibit any neurological signs (day 8 or 9; N = 3), and PyXL-infected mice with hyperparasitemia (day 5; N = 3) were immunolabeled with specific antibodies the TJ proteins claudin-5, occludin, and ZO-1. Multiple confocal microscopy images per experimental condition were acquired and the overall TJ protein-specific fluorescence emission of the microvascular endothelium quantified with ImageJ. No significant reduction in TJ protein expression was detected under the different infection and treatment conditions compared to uninfected control mice (N = 3).
(TIF)

Table S1 **Number of mice examined by IVM.** CBA/CaJ mice were infected with PbA, PyXL, or no parasites, and subjected to craniotomy, and surgically prepared for IVM. PbA infected mice were analyzed at the time of ECM (day 6–8), before the appearance of neurological signs (day 5), or after the window of ECM development had passed (day 9). PyXL infected mice were examined at the parasitemia exceeding 50%. Other mice were treated daily with FTY720 starting one day before infection with PbA and examined by IVM on day 8 or 9.
(DOCX)

Table S2 **CD8+ T cell density in PCV.** CBA/CaJ mice were infected with PbA, PyXL, or no parasites, and subjected to craniotomy, and prepared for IVM. PbA infected mice were analyzed at the time of ECM (day 6–8), before the appearance of neurological signs (day 5), or after the window of ECM development had passed (day 9). PyXL infected mice were examined at the parasitemia exceeding 50%. CD8+ T cells were labeled by intravenous inoculation of PE-conjugated anti-CD8a. Significantly larger numbers of CD8+ T cells were recruited to PCV from mice with ECM compared to HP (* = p<0.05). No CD8+ T cells were found in the cortical microvasculature of PbA infected mice on day 5, in PbA infected mice that survived the critical period of ECM development without exhibiting neurological signs (day 9), or in uninfected control mice. The data represent the mean cell density/mm^2^ ± SEM. The significance (PbA vs. PyXL) was determined by 1-way ANOVA. See also **Figures 2** **and** **3** and **Videos S6 and S7**.
(DOCX)

Table S3 **CD4+ T cell density in PCV.** CBA/CaJ mice were infected with PbA, PyXL, or no parasites, and subjected to craniotomy, and prepared for IVM. PbA infected mice were analyzed at the time of ECM (day 6–8), before the appearance of neurological signs (day 5), or after the window of ECM development had passed (day 9). PyXL infected mice were examined at the parasitemia exceeding 50%. Depending on the fluorescence of the parasites, CD8+ T cells were labeled by intravenous inoculation of PE- or eFluor 450-or PE-conjugated anti-CD4. Significantly larger numbers of CD4+ T cells were recruited to PCV from mice with ECM compared to HP (* = p<0.05). No CD4+ T cells were found in the cortical microvasculature of PbA infected mice on day 5, in PbA infected mice that survived the critical period of ECM development without exhibiting neurological signs (day 9), or in uninfected control mice. The data represent the mean cell density/mm^2^ ± STD. The significance (PbA vs. PyXL) was determined by 1-way ANOVA. See also **Figures 2** **and** **3** and **Videos S8 and S9**.
(DOCX)

Table S4 **Neutrophil density in PCV.** CBA/CaJ mice were infected with PbA, PyXL, or no parasites, and subjected to craniotomy, and prepared for IVM. PbA infected mice were analyzed at the time of ECM (day 6–8), before the appearance of neurological signs (day 5), or after the window of ECM development had passed (day 9). PyXL infected mice were examined at the parasitemia exceeding 50%. CD8+ T cells were labeled by intravenous inoculation of eFluor 450-conjugated GR-1. Significantly larger numbers of neutrophils were recruited to PCV from mice with ECM compared to HP (* = p<0.05). No neutrophils were found in the cortical microvasculature of PbA infected mice on day 5, in PbA infected mice that survived the critical period of ECM development without exhibiting neurological signs (day 9), or in uninfected control mice. The data represent the mean cell density/mm^2^ ± SEM. The significance (PbA vs. PyXL) was determined by 1-way ANOVA. See also **Figures 2** **and** **3** and **Videos S10 and S11**.
(DOCX)

Table S5 **Monocyte density in PCV.** CBA/CaJ mice were infected with PbA, PyXL, or no parasites, and subjected to craniotomy, and prepared for IVM. PbA infected mice were analyzed at the time of ECM (day 6–8), before the appearance of neurological signs (day 5), or after the window of ECM development had passed (day 9). PyXL infected mice were examined at the parasitemia exceeding 50%. CD8+ T cells were labeled by intravenous inoculation of PE-conjugated anti-CD14. Significantly larger numbers of monocytes were recruited to PCV from mice with ECM compared to HP (* = p<0.05). No monocytes were found in the cortical microvasculature of PbA infected mice on day 5, in PbA infected mice that survived the critical period of ECM development without exhibiting neurological signs (day 9), or in uninfected control mice. The data represent the mean cell density/mm^2^ ± SEM. The significance (PbA vs. PyXL) was determined by 1-way ANOVA. See also **Figures 2** **and** **3** and **Videos S12 and S13**.
(DOCX)

Table S6 **Macrophage density in PCV.** CBA/CaJ mice were infected with PbA, PyXL, or no parasites, and subjected to craniotomy, and prepared for IVM. PbA infected mice were analyzed at the time of ECM (day 6–8), before the appearance of neurological signs (day 5), or after the window of ECM development had passed (day 9). PyXL infected mice were examined at the parasitemia exceeding 50%. CD8+ T cells were labeled by intravenous inoculation of eFluor 450-conjugated anti-CD11b. Significantly larger numbers of macrophages were recruited to PCV from mice with ECM compared to HP (* = p<0.05). No macrophages were found in the cortical microvasculature of PbA infected mice on day 5, in PbA infected mice that survived the critical period of ECM development without exhibiting neurological signs (day 9), or in uninfected control mice. The data represent the mean cell density/mm^2^ ± SEM. The significance (PbA vs. PyXL) was determined by 1-way ANOVA. See also **Figures 2** **and** **3** and **Videos S14 and S15**.
(DOCX)

Table S7 **Leukocyte accumulation in the brain.** PbA-infected mice with ECM (day 6–8; N = 3), PbA-infected/FTY720-treated mice without neurological signs (day 8 or 9; N = 3), and PyXL-infected mice with HP (N = 3) were vascularly perfused prior to isolation of leukocytes from the cerebrum and cerebellum. Adherent cells were isolated and subjected to flow cytometry. CD45+ F4/80+ cells represent the entire macrophage population of the brain, while the CD45^hi^ F4/80+ macrophages are predominantly blood-derived. The data represent the average cell number per 50,000 events ± STD. Statistical analysis was performed using a 1-way ANOVA followed by Tukey's test for multiple comparisons. See also **Figure 3**.
(DOCX)

Table S8 **CD45^hi^ and CD45^lo^ leukocyte subsets.** PbA-infected mice with ECM (day 6–8; N = 3), PbA-infected/FTY720-treated mice without neurological signs (day 8 or 9; N = 3), and PyXL-infected mice with HP (day 5: N = 3) were vascularly perfused prior to isolation of leukocytes from the cerebrum and cerebellum. Adherent cells were isolated and subjected to flow cytometry. The data represent the average cell number per 50,000 events ± STD. Statistical analysis was performed using a 1-way ANOVA followed by Tukey's test for multiple comparisons. See also **Figure 4**.
(DOCX)

Table S9 **ICAM-1 positive CD45^hi^ and CD45^lo^ leukocyte subsets.** PbA-infected mice with ECM (day 6–8; N = 5), PbA-infected/FTY720-treated mice without neurological signs (day 8 or 9; N = 3), and PyXL-infected mice with HP (day 5: N = 3) were vascularly perfused prior to isolation of leukocytes from the cerebrum and cerebellum. Adherent cells were isolated and subjected to flow cytometry. The data represent the average cell number per 50,000 events ± STD. Statistical analysis was performed using a 1-way ANOVA followed by Tukey's test for multiple comparisons. See also **Figure 4**.
(DOCX)

Table S10 **CD69-positive CD45^hi^ and CD45^lo^ leukocyte subsets.** PbA-infected mice with ECM (day 6–8; N = 3), PbA-infected/FTY720-treated mice without neurological signs (day 8 or 9; N = 3), and PyXL-infected mice with HP (day 5: N = 3) were vascularly perfused prior to isolation of leukocytes from the cerebrum and cerebellum. Adherent cells were isolated and subjected to flow cytometry. The data represent the average cell number per 50,000 events ± STD. Statistical analysis was performed using a 1-way ANOVA followed by Tukey's test for multiple comparisons. See also **Figure 4**.
(DOCX)

Table S11 **GrB-positive CD45^hi^ and CD45^lo^ leukocyte subsets.** PbA-infected mice with ECM (day 6–8; N = 3), PbA-infected/FTY720-treated mice without neurological signs (day 8 or 9; N = 3), and PyXL-infected mice with HP (day 5: N = 3) were vascularly perfused prior to isolation of leukocytes from the cerebrum and cerebellum. Adherent cells were isolated and subjected to flow cytometry. The data represent the average cell number per 50,000 events ± STD. Statistical analysis was performed using a 1-way ANOVA followed by Tukey's test for multiple comparisons. See also **Figure 4**.
(DOCX)

Table S12 **Phenotype of CD45^hi^ CD8+ T cells.** PbA-infected mice with ECM (day 6–8; N = 3), PbA-infected/FTY720-treated mice without neurological signs (day 8 or 9; N = 3), and PyXL-infected mice with HP (day 5: N = 3) were vascularly perfused prior to isolation of leukocytes from the cerebrum and cerebellum. Adherent cells were isolated and subjected to flow cytometry. The data represent the average cell number per 50,000 events ± STD. Statistical analysis was performed using a 1-way ANOVA followed by Tukey's test for multiple comparisons. See also **Figure S4**.
(DOCX)

Table S13 **Endothelial ICAM-1 expression in the cortical microvasculature.** Two PbA-infected mice with ECM (day 6–8), 2 PyXL-infected mice with HP (day 5), and 2 uninfected control mice were intravenously inoculated with PE-conjugated rat anti-mouse CD54 and subjected to IVM. Relative fluorescence emission was quantified as described in Materials & Methods and expressed as average ± STD. Statistical analysis was performed using 1-way ANOVA followed by Tukey's test for multiple comparisons. See also **Figure 5**.
(DOCX)

Table S14 **Number of ICAM-1+ F4/80+ macrophages.** PbA-infected mice with ECM (day 6–8; N = 3), PbA-infected/FTY720-treated mice without neurological signs (day 8 or 9; N = 3), and PyXL-infected mice with HP (day 5: N = 3) were vascularly perfused prior to isolation of leukocytes from the cerebrum and cerebellum. Adherent cells were isolated and subjected to flow cytometry. The data represent the average cell number per 50,000 events ± STD. T-test was used to determine significances. See also **Figure 6A and 6B**.
(DOCX)

Table S15 **ICAM-1 expression level on F4/80+ macrophages.** PbA-infected mice with ECM (day 6–8; N = 3), PbA-infected/FTY720-treated mice without neurological signs (day 8 or 9; N = 3), and PyXL-infected mice with HP (day 5: N = 3) were vascularly perfused prior to isolation of leukocytes from the cerebrum and cerebellum. Adherent cells were isolated and subjected to flow cytometry. The data represent the median cell number per 50,000 events ± STD. Statistical analysis was performed using a 1-way ANOVA followed by Tukey's Test for multiple comparisons. See also **Figure 6C**.
(DOCX)

Table S16 **Tight junction protein expression in the cerebral cortex and cerebellum.** Cryostat sections of brain tissue from PbA-infected CBA/CaJ mice with ECM (day 6–8; N = 4), PbA-infected and FTY720-treated mice that did not exhibit any neurological signs (day 8 or 9; N = 3), and PyXL-infected mice with HP (day 5; N = 3) were immunolabeled with specific antibodies the TJ proteins claudin-5, occludin, and ZO-1. Confocal microscopy images (3–4 per experimental condition) were acquired under identical conditions. Images were imported into ImageJ for quantification of the average TJ protein-specific fluorescence emission with the threshold set to Triangle. The data represent average fluorescence intensity ± SD in the entire field of observation. Significant differences (* = *P*<0.05) in TJ protein expression under the different infection and treatment conditions were determined by t-test in relation to uninfected control mice (N = 3). See also **Figure S6 and S7**.
(DOCX)

Video S1 **ECM is associated with a significant reduction in the venous blood flow.** Intravital microscopy of a PbA-infected CBA/CaJ mouse injected with Evans blue (red) upon development of ECM symptoms. Arrested or crawling leukocytes (dark circles) prevent access to the endothelium so that the marginal zone of the postcapillary venule lumen lacks blood cells traveling in the bloodstream (dark streaks). Note the extensive leakage of Evans blue from the cortical postcapillary venules in the surrounding tissue. Scale bar = 20 µm.
(AVI)

Video S2 **ECM is associated with a significant reduction in the venous blood flow.** Intravital microscopy of a PbA-infected CBA/CaJ mouse injected with Evans blue (red) upon development of ECM symptoms. Arrested leukocytes (dark circles) prevent access to the endothelium so that the marginal zone of the postcapillary venule lumen lacks blood cells traveling in the bloodstream (dark streaks). Evans blue has leaked from the cortical postcapillary venules in the surrounding tissue. Scale bar = 20 µm.
(AVI)

Video S3 **The arteriolar microrheology remains unaltered during ECM.** Intravital microscopy of a PbA-infected CBA/CaJ mouse showing normal blood flow in a cortical arteriole (vessel running horizontally). Note that the entire microvascular lumen is used for the blood flow. The vascular lumen is visualized with Evans blue (red), while blood cells are negatively stained (dark streaks). Scale bar = 20 µm.
(AVI)

Video S4 **The venous microrheology remains unaltered during hyperparasitemia.** Intravital microscopy of a PyXL infected CBA/CaJ mouse exhibiting normal blood flow in a cortical postcapillary venules. Note that the entire microvascular lumen is used for the blood flow. The vascular lumen is visualized with Evans blue (red), while blood cells are negatively stained (dark streaks). Scale bar = 20 µm.
(AVI)

Video S5 **Normal venous microrheology of uninfected control mice.** Intravital microscopy of an uninfected control CBA/CaJ mouse exhibiting normal blood flow in a cortical postcapillary venule. Note that the entire microvascular lumen is used for the blood flow. The vascular lumen is visualized with Evans blue (red), while blood cells are negatively stained (dark streaks). Scale bar = 20 µm.
(AVI)

Video S6 **CD8+ T cell behavior during ECM.** Intravital microscopy of a PbA infected CBA/CaJ mouse with ECM showing a large number of CD8+ T cells (green surface label) crawling along the endothelium of a cortical postcapillary venules with a typical leading edge and trailing uropod (arrow). CD8+ T cells were surface-labeled by intravenous inoculation of PE-conjugated anti-CD8a. The vascular lumen was visualized with Evans blue (red). Scale bar = 20 µm.
(AVI)

Video S7 **CD8+ T cell behavior during hyperparasitemia.** Intravital microscopy of a PyXL infected CBA/CaJ mouse with hyperparasitemia showing a small number of CD8+ T cells (green surface label) crawling along the endothelium of a cortical postcapillary venules. CD8+ T cells were labeled by intravenous inoculation of eFluor 450-conjugated anti-CD8a (false-colored in green). The vascular lumen was visualized with Evans blue (red). Scale bar = 20 µm.
(AVI)

Video S8 **CD4+ T cell behavior during ECM.** Intravital microscopy of a PbA infected CBA/CaJ mouse with ECM showing CD4+ T cells (green surface label) arrested on the endothelium of a cortical postcapillary venules. CD4+ T cells were labeled by intravenous inoculation of PE-conjugated anti-CD4+. The vascular lumen was visualized with Evans blue (red). The numerous large leukocytes (dark circles) represent most likely monocytes and macrophages. Scale bar = 20 µm.
(AVI)

Video S9 **CD4+ T cell behavior during hyperparasitemia.** Intravital microscopy of a PyXL infected CBA/CaJ mouse with hyperparasitemia showing CD4+ T cells (green surface label) arrested on the endothelium of a cortical postcapillary venules. CD4+ T cells were labeled by intravenous inoculation of PE-conjugated anti-CD4+. The vascular lumen was visualized with Evans blue (red). Scale bar = 20 µm.
(AVI)

Video S10 **Neutrophil behavior during ECM.** Intravital microscopy of a PbA infected CBA/CaJ mouse with ECM showing large numbers of neutrophils (green surface label) arrested on the endothelium of a cortical postcapillary venules. Neutrophils were labeled by intravenous inoculation of eFluor 450-conjugated GR-1 (false-colored in green). The vascular lumen was visualized with Evans blue (red). Scale bar = 20 µm.
(AVI)

Video S11 **Neutrophil behavior during hyperparasitemia.** Intravital microscopy of a PyXL infected CBA/CaJ mouse with hyperparasitemia showing a small number of neutrophils (green surface label) arrested on the endothelium of a cortical postcapillary venules. Neutrophils were labeled by intravenous inoculation of eFluor 450-conjugated GR-1 (false-colored in green). The vascular lumen was visualized with Evans blue (red). Scale bar = 20 µm.
(AVI)

Video S12 **Monocyte behavior during ECM.** Intravital microscopy of a PbA infected CBA/CaJ mouse with ECM showing monocytes (green surface label) arrested on the endothelium of a cortical postcapillary venules. Monocytes were labeled by intravenous inoculation of PE-conjugated anti-CD14. Note that the fluorescent marker also labels the endothelium of the postcapillary venules. The vascular lumen was visualized with Evans blue (red). The numerous large leukocytes (dark circles) represent most likely monocytes and macrophages. Scale bar = 20 µm.
(AVI)

Video S13 **Monocyte behavior during hyperparasitemia.** Intravital microscopy of a PyXL infected CBA/CaJ mouse with hyperparasitemia showing a monocyte (green) arrested on the endothelium of a cortical postcapillary venules. The mouse was intravenously inoculated with PE-conjugated anti-CD14. Note that CD14 is not expressed on the endothelium of the postcapillary venules during hyperparasitemia. The vascular lumen was visualized with Evans blue (red). Scale bar = 20 µm.
(AVI)

Video S14 **Macrophage behavior during ECM.** Intravital microscopy of a PbA infected CBA/CaJ mouse with ECM showing macrophages (green surface label) arrested on the endothelium of a cortical postcapillary venules. Macrophages were labeled by intravenous inoculation of Pacific blue-conjugated anti-CD11b. The vascular lumen was visualized with Evans blue (red). Scale bar = 20 µm.
(AVI)

Video S15 **Macrophage behavior during hyperparasitemia.** Intravital microscopy of a PyXL infected CBA/CaJ mouse with hyperparasitemia showing macrophages (green surface label) arrested on the endothelium of a cortical postcapillary venules. Macrophages were labeled by intravenous inoculation of Pacific blue-conjugated anti-CD11b (false-colored in green). The vascular lumen was visualized with Evans blue (red). Scale bar = 20 µm.
(AVI)

Video S16 **Monocyte behavior after FTY720 treatment.** Intravital microscopy stack of a PbA infected and FTY720-treated CBA/CaJ mouse without ECM showing monocytes (green surface label) arrested on the endothelium of a cortical postcapillary venules. Monocytes were labeled by intravenous inoculation of PE-conjugated anti-CD14. The endothelium of the postcapillary venules is CD14-positive despite the FTY720 treatment. Note that the smaller arteriole on the left is CD14-negative. The vascular lumen was visualized with Evans blue (red). Scale bar = 20 µm.
(AVI)

Video S17 **ICAM-1 is upregulated during ECM.** Intravital microscopy stack showing that ICAM-1 (gray) is expressed on the surface of postcapillary venules and, to a lesser degree, also on arteriolar endothelia (day 6–8 after infection with PbA). Note that some arrested leukocytes also express ICAM-1 (gray surface label). Scale bar = 20 µm.
(AVI)

Video S18 **ICAM-1 is upregulated during hyperparasitemia.** Intravital microscopy stack showing that ICAM-1 (gray) is expressed on the surface of postcapillary venules and, to a lesser degree, also on arteriolar endothelia (day 5 after infection with PyXL). Note that the number of arrested ICAM-1 expressing leukocytes (gray surface label) is lower during hyperparasitemia compared to ECM (**Video S12**). Scale bar = 20 µm.
(AVI)

Video S19 **ICAM-1 expression in an uninfected control mouse.** Intravital microscopy stack showing a lower level of ICAM-1 (gray) expression on the surface of postcapillary venules compared to mice with ECM (**Video S12**) or hyperparasitemia (**Video S13**). ICAM-1 expressing leukocytes are absent. Scale bar = 20 µm.
(AVI)

Video S20 **ECM is associated with platelet arrest and P-selectin expression.** CBA/CaJ mouse infected with PbA and inoculated with eFluor 450-conjugated anti-CD41 at the time of ECM. The intravital microscopy movie shows small clusters of marginalized platelets (blue) whose surface is positive for PE-conjugated anti-CD62p (green). The lumen of the cortical postcapillary venules is visualized with Evans blue (red). Scale bar = 20 µm.
(AVI)

Video S21 **Mice with ECM exhibit strings of platelets in postcapillary venules.** At the time of ECM, a PbA-infected CBA/CaJ mouse that was inoculated with eFluor 450-conjugated anti-CD41 (blue). A postcapillary venule contains a string of platelets (blue), which appears to be attached to a cluster of marginalized platelets. Scale bar = 20 µm.
(AVI)

Video S22 **Hyperparasitemia is not associated with platelet arrest or P-selectin expression.** Intravital microscopy of a CBA/CaJ mouse infected with PyXL-RFP iRBC shows eFluor 450-labeled platelets (blue) circulating at blood velocity. There is no evidence for P-selectin labeling, although the mouse was inoculated with a PE-conjugated specific antibody (green). The lumen of the cortical PVC is visualized with Evans blue (red). Scale bar = 20 µm.
(AVI)
