# Supplementary figures and images for: Experimental Cerebral Malaria Pathogenesis—Hemodynamics at the Blood Brain Barrier
Source: PLoS Pathog. 2014 Dec 4;10(12):e1004528. doi: 10.1371/journal.ppat.1004528 (PMC4256476; doi:10.1371/journal.ppat.1004528)

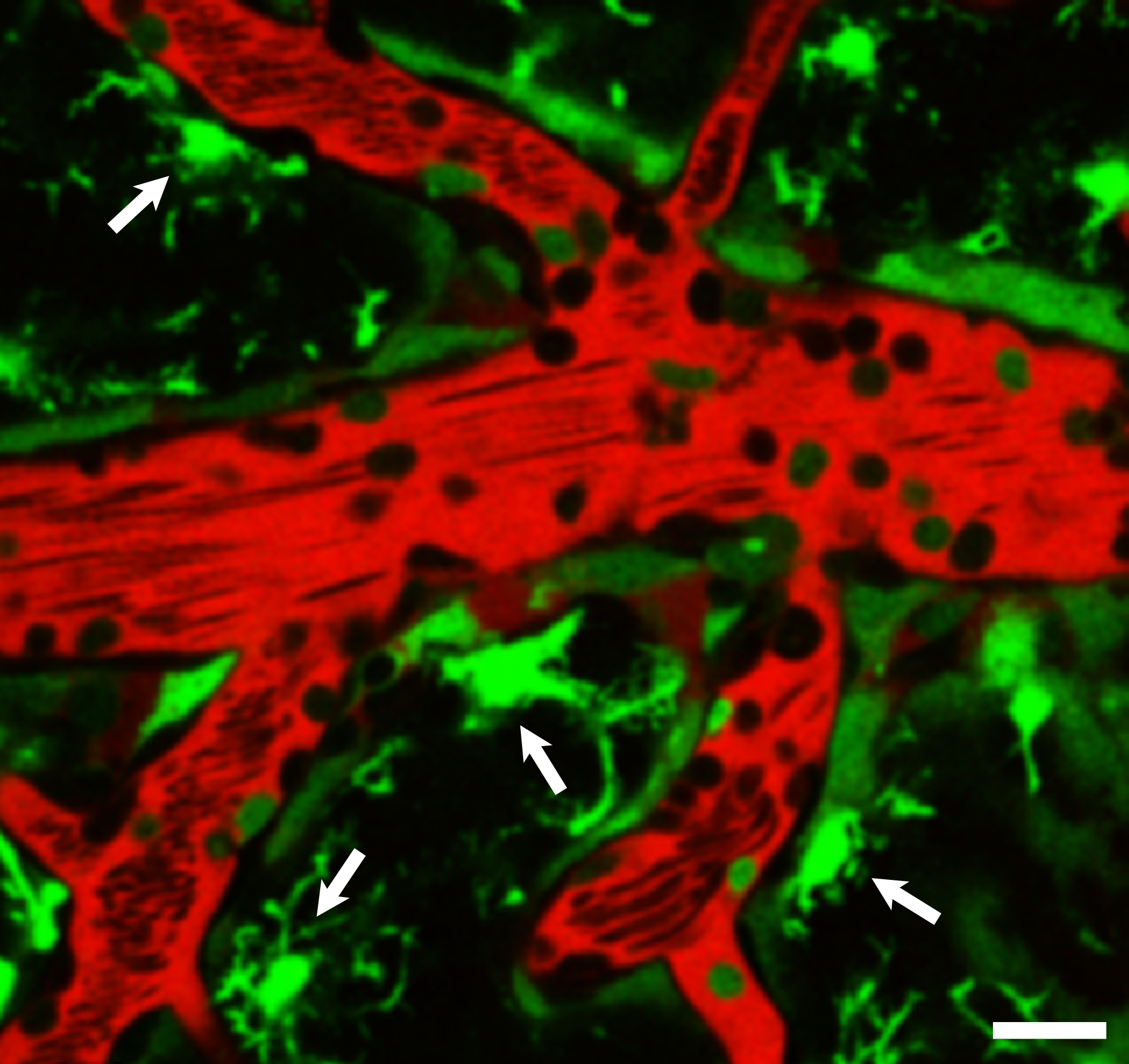

Supplement: Figure S1 — Confocal intravital microscopy of the cortical microvasculature. Intravital microscopy of a PbA-infected CX3CR1GFP/+ mouse with ECM shows that the postcapillary venules, capillaries, and arterioles analyzed in this study are embedded in green fluorescent microglia (arrows) and thus located in the cerebral cortex, i.e. below the layer of pial microvessels. Evans blue (red) visualizes the microvascular lumen, while blood cells are negatively stained (black). Leukocytes crawl slowly (large black circles), while RBCs move at bloodstream velocity and appear as dark streaks. Note that the vascular marker has leaked into the perivascular space. Scale bar = 50 µm. (TIF) [file ppat.1004528.s001.tif]

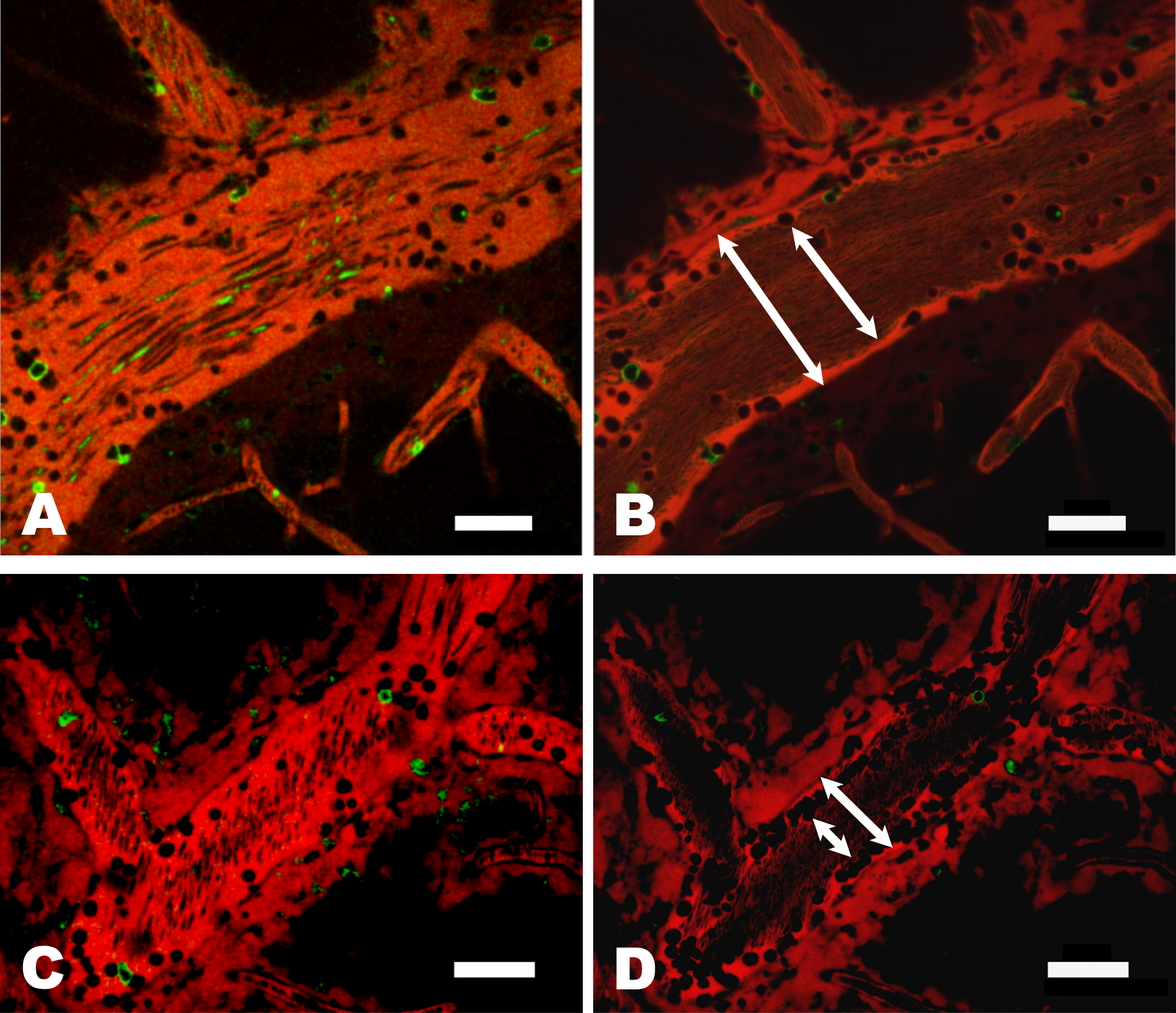

Supplement: Figure S2 — Visualization of the luminal restriction of postcapillary venules during ECM. Examples of intravital movies and minimal projections of postcapillary venules from PbA-infected mice with ECM. A, C) Individual frames show postcapillary venules containing blood cells (dark circles or streaks) that are distorted due to the relatively slow confocal scan speed compared to the much faster blood flow. B, D) Minimal projections of the movies reveal that the functional vessel diameter (short arrows), i.e. the perfused portion of the vessel, is considerably reduced compared to the entire vessel diameter (long arrows). Visualization of the vascular lumen with Evans blue reveals a red zone along the endothelium of postcapillary venules that is devoid of RBC (dark). Scale bars = 20 µm. See Video S1 and 2 for the corresponding dynamic data. (TIF) [file ppat.1004528.s002.tif]

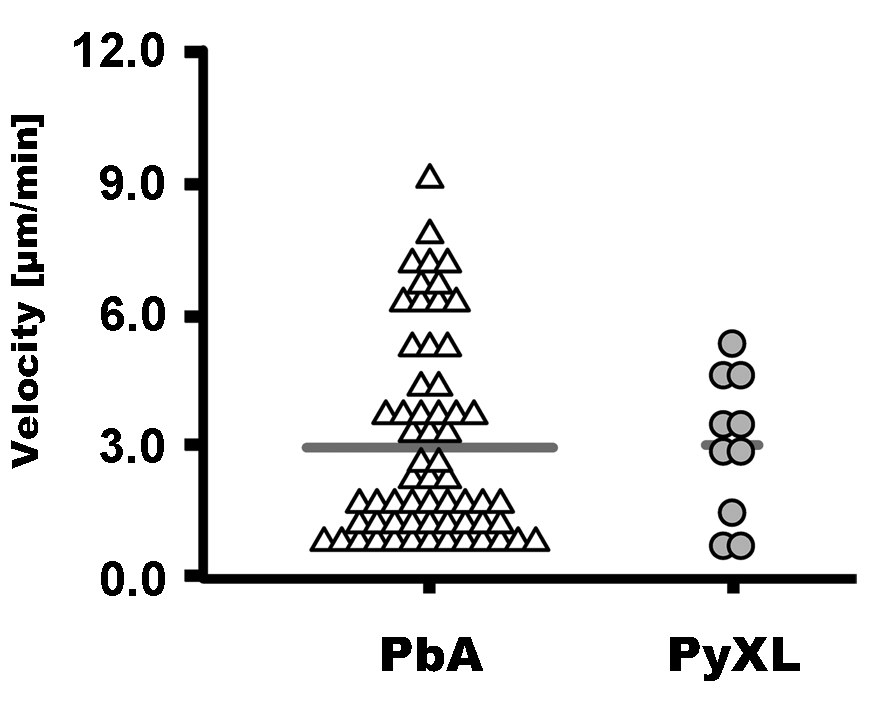

Supplement: Figure S3 — CD8+ T cell velocity during ECM and hyperparasitemia. CBA/CaJ mice infected with PbA or PyXL were inoculated with PE or eFluor 450-conjugated anti-CD8+a at the time of ECM or hyperparasitemia, respectively, and velocity and density of CD8+ T cells was determined by off-line analysis of intravital microscopy time sequences and 3D stacks, respectively. Mean velocity of intravascular CD8+ T cells during ECM (PbA) and hyperparasitemia (PyXL). The data represent the mean ± SEM of 61 cells from 5 PbA-infected and 10 cells from 5 PyXL-infected mice. Significance was calculated with 1-way ANOVA. (TIF) [file ppat.1004528.s003.tif]

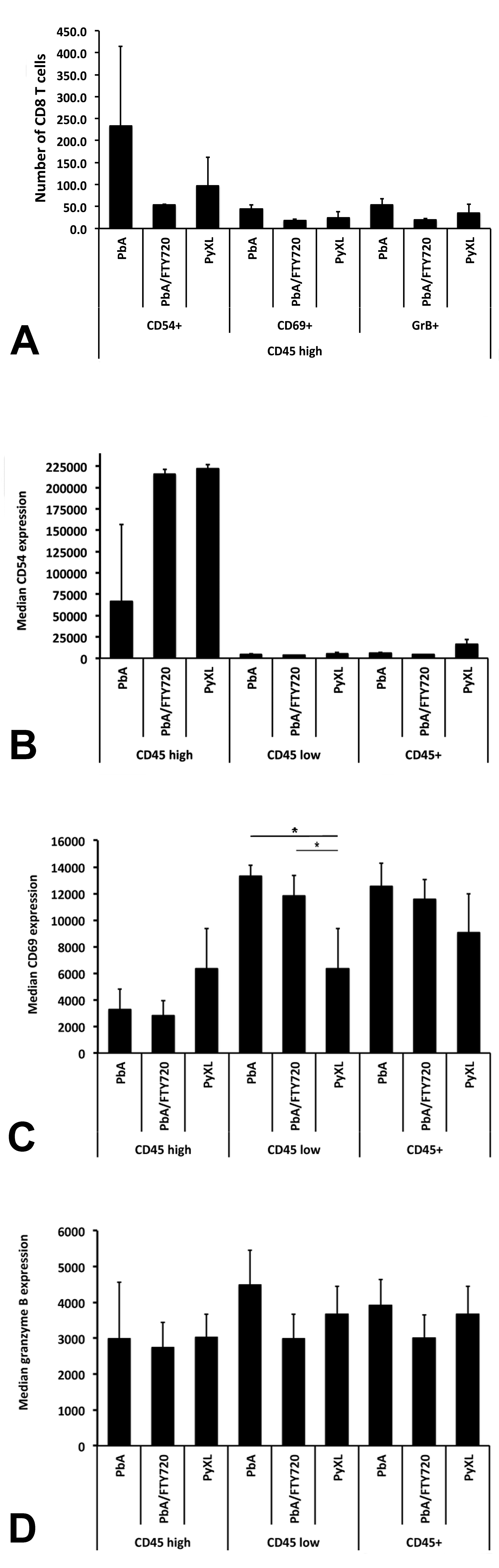

Supplement: Figure S4 — ICAM-1, CD69, and GrB expression in CD8+ T cells. Leukocytes were isolated from the brains of PbA-infected, PbA-infected/FTY720-treated, and PyXL-infected mice. A) Flow cytometry reveals that FTY720 treatment reduces the ECM-associated accumulation of ICAM-1+ CD69+ GrB+ CD45hi CD8+ T cells in the brain of PbA-infected mice to levels similar to those found in PyXL-infected mice with hyperparasitemia. The data are based on groups of at least 3 mice per experimental condition. Significance was determined with 1-way ANOVA followed by Tukey's test for multiple comparisons. See Table S12 for details. B–D) Flow cytometry revealed no significant difference in the median expression levels of ICAM-1 (CD54) (B), CD69 (C), or GrB (D) in the CD45hi subset of CD8+ T cells compared to PyXL-infected or PbA-infected/FTY720-treated mice. Data are based on 3 mice per group. Significance (*, P<0.05) was determined with 1-way ANOVA followed by Tukey's test for multiple comparisons. (TIF) [file ppat.1004528.s004.tif]

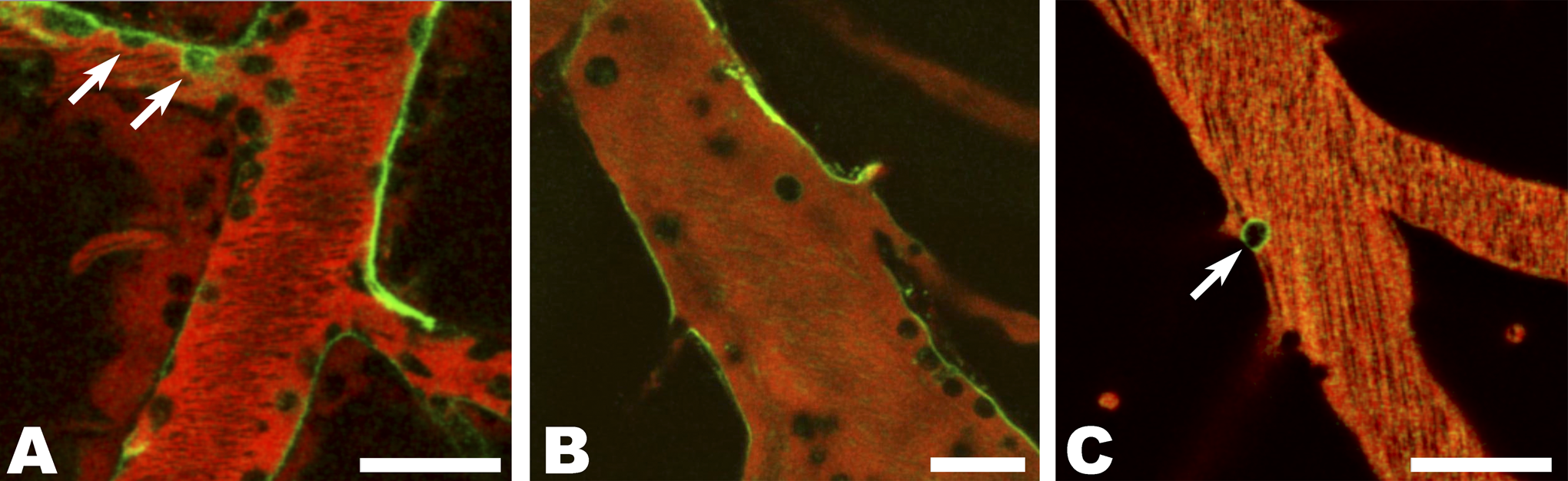

Supplement: Figure S5 — Effect of FTY720 on endothelial CD14 expression. Postcapillary venule endothelia of PbA-infected mice are positive for CD14 at the time of ECM (green outline) (A). FTY720 treatment does not prevent endothelial CD14 expression in postcapillary venules (green outline) (B). PyXL-infected mice do not exhibit endothelial CD14 at the time of hyperparasitemia (C). Mice were inoculated with Evans blue (red) to visualize the vascular lumen and PE-conjugated anti-CD14. Note that monocytes (green open circles) are also CD14 positive (arrows in A and C). See Videos S12, S13, and S16 for the corresponding dynamic data. (TIF) [file ppat.1004528.s005.tif]

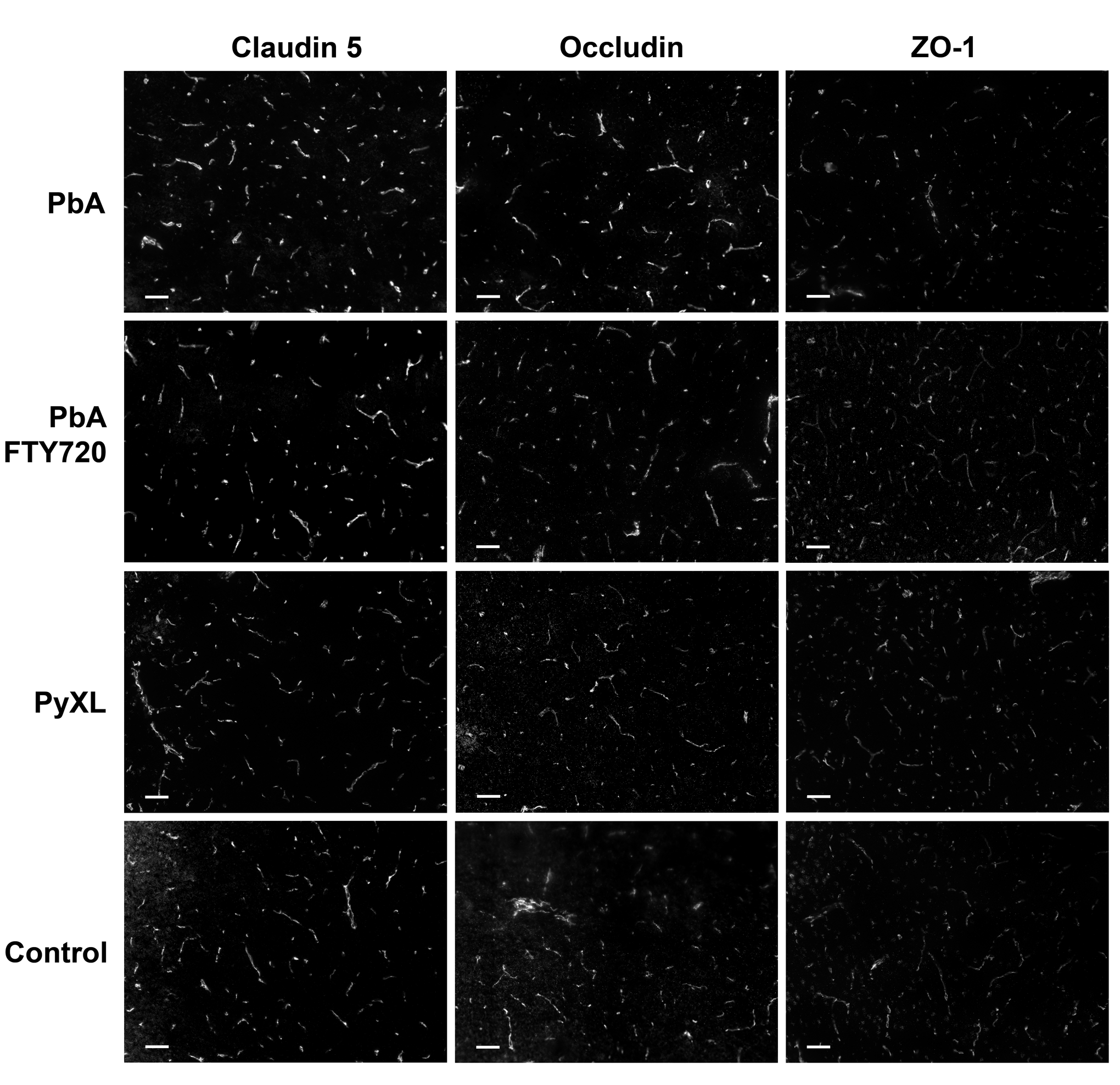

Supplement: Figure S6 — TJ protein expression in the cerebral cortex. Cryostat sections of the brains of PbA-infected CBA/CaJ mice with ECM (day 6–8; N = 4), PbA-infected and FTY720-treated mice that did not exhibit any neurological signs (day 8 or 9; N = 3), and PyXL-infected mice with hyperparasitemia (day 5; N = 3) were immunolabeled with specific antibodies the TJ proteins claudin-5, occludin, and ZO-1. Multiple confocal microscopy images per experimental condition were acquired and the overall TJ protein-specific fluorescence emission was quantified with ImageJ. No significant reduction in TJ protein expression was detected under the different infection and treatment conditions compared to uninfected control mice (N = 3). (TIF) [file ppat.1004528.s006.tif]

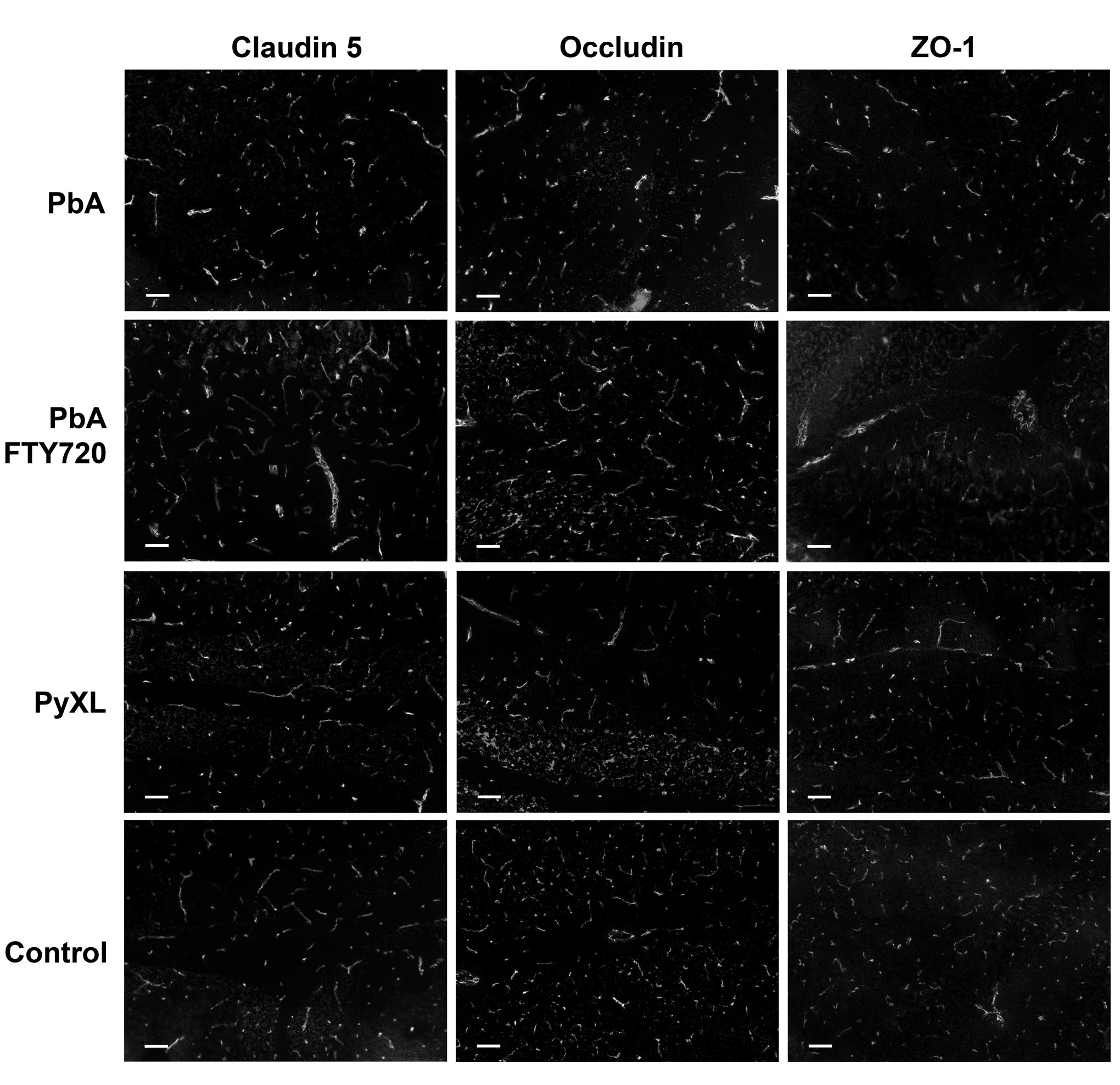

Supplement: Figure S7 — TJ protein expression in the cerebellum. Cryostat sections of the brains of PbA-infected CBA/CaJ mice with ECM (day 6–8; N = 4), PbA-infected and FTY720-treated mice that did not exhibit any neurological signs (day 8 or 9; N = 3), and PyXL-infected mice with hyperparasitemia (day 5; N = 3) were immunolabeled with specific antibodies the TJ proteins claudin-5, occludin, and ZO-1. Multiple confocal microscopy images per experimental condition were acquired and the overall TJ protein-specific fluorescence emission of the microvascular endothelium quantified with ImageJ. No significant reduction in TJ protein expression was detected under the different infection and treatment conditions compared to uninfected control mice (N = 3). (TIF) [file ppat.1004528.s007.tif]
