# Supplementary material for: Experimental Cerebral Malaria Pathogenesis—Hemodynamics at the Blood Brain Barrier
Source: PLoS Pathog. 2014 Dec 4;10(12):e1004528. doi: 10.1371/journal.ppat.1004528 (PMC4256476; doi:10.1371/journal.ppat.1004528)
Supplement: Table S1 — Number of mice examined by IVM. CBA/CaJ mice were infected with PbA, PyXL, or no parasites, and subjected to craniotomy, and surgically prepared for IVM. PbA infected mice were analyzed at the time of ECM (day 6–8), before the appearance of neurological signs (day 5), or after the window of ECM development had passed (day 9). PyXL infected mice were examined at the parasitemia exceeding 50%. Other mice were treated daily with FTY720 starting one day before infection with PbA and examined by IVM on day 8 or 9. (DOCX) [file ppat.1004528.s008.docx]

**Table S1. Number of mice examined by IVM**

| **Infection / treatment** | **Day of imaging** | **Signs** | **Number of mice analyzed** |
| --- | --- | --- | --- |
|  |  |  |  |
|  |  |  |  |
| **PbA** | 6-8 | ECM | 78 |
| **PbA** | 5 | Prior to ECM | 18 |
| **PbA** | 9 | Failed to develop ECM | 15 |
| **PbA / FTY720** | 8-9 | Failed to develop ECM | 4 |
| **PbA / FTY720** | 8-9 | ECM despite treatment | 8 |
| **PyXL** | 5 | Hyperparasitemia | 62 |
| **Uninfected** | N/A | None | 25 |

CBA/CaJ mice were infected with PbA, PyXL, or no parasites, and subjected to craniotomy, and surgically prepared for IVM. PbA infected mice were analyzed at the time of ECM (day 6-8), before the appearance of neurological signs (day 5), or after the window of ECM development had passed (day 9). PyXL infected mice were examined at the parasitemia exceeding 50%. Other mice were treated daily with FTY720 starting one day before infection with PbA and examined by IVM on day 8 or 9.
