# Supplementary material for: Experimental Cerebral Malaria Pathogenesis—Hemodynamics at the Blood Brain Barrier
Source: PLoS Pathog. 2014 Dec 4;10(12):e1004528. doi: 10.1371/journal.ppat.1004528 (PMC4256476; doi:10.1371/journal.ppat.1004528)
Supplement: Table S6 — Macrophage density in PCV. CBA/CaJ mice were infected with PbA, PyXL, or no parasites, and subjected to craniotomy, and prepared for IVM. PbA infected mice were analyzed at the time of ECM (day 6–8), before the appearance of neurological signs (day 5), or after the window of ECM development had passed (day 9). PyXL infected mice were examined at the parasitemia exceeding 50%. CD8+ T cells were labeled by intravenous inoculation of eFluor 450-conjugated anti-CD11b. Significantly larger numbers of macrophages were recruited to PCV from mice with ECM compared to HP (* = p<0.05). No macrophages were found in the cortical microvasculature of PbA infected mice on day 5, in PbA infected mice that survived the critical period of ECM development without exhibiting neurological signs (day 9), or in uninfected control mice. The data represent the mean cell density/mm2 ± SEM. The significance (PbA vs. PyXL) was determined by 1-way ANOVA. See also Figures 2 and 3 and Videos S14 and S15. (DOCX) [file ppat.1004528.s013.docx]

**Table S6. Macrophage density in PCV**

|  | **Overall density [cells/mm^2^]**  **Total number of cells** | **Vascular diameter** **[µm]**  **Number of PCV analyzed** | **Number of mice analyzed** |
| --- | --- | --- | --- |
|  |  |  |  |
|  |  |  |  |
| **PbA / ECM**  **Day 6-8** | 484.1 ± 13.1  N = 96 | 31.8 ± 16.2  N = 78 | 3 |
| **PbA / no ECM**  **Day 5** | 0 | 30.8 ± 12.2  N = 10 | 1 |
| **PbA / no ECM**  **(V-1 positive control mice (1 mouse). of the rior to ECM (2 mice) or in ECM surviovors (not done). ected control mice. Day 9** | 0 | 34.6 ± 17.2  N = 10 | 1 |
| **PyXL / HP**  **Day 5** | 110.2 ± 17.4  N = 31 | 34.3 ± 15.2  N = 40 | 5 |
| **Uninfected** | 0 | 26.8 ± 11.9  N = 45 | 1 |

CBA/CaJ mice were infected with PbA, PyXL, or no parasites, and subjected to craniotomy, and prepared for IVM. PbA infected mice were analyzed at the time of ECM (day 6-8), before the appearance of neurological signs (day 5), or after the window of ECM development had passed (day 9). PyXL infected mice were examined at the parasitemia exceeding 50%. CD8+ T cells were labeled by intravenous inoculation of eFluor 450-conjugated anti-CD11b. Significantly larger numbers of macrophages were recruited to PCV from mice with ECM compared to HP (* = p < 0.05). No macrophages were found in the cortical microvasculature of PbA infected mice on day 5, in PbA infected mice that survived the critical period of ECM development without exhibiting neurological signs (day 9), or in uninfected control mice. The data represent the mean cell density/mm^2^ ± SEM. The significance (PbA vs. PyXL) was determined by 1-way ANOVA. See also **Figures 2 and 3** and **Videos S14 and S15**.
