# Supplementary material for: Experimental Cerebral Malaria Pathogenesis—Hemodynamics at the Blood Brain Barrier
Source: PLoS Pathog. 2014 Dec 4;10(12):e1004528. doi: 10.1371/journal.ppat.1004528 (PMC4256476; doi:10.1371/journal.ppat.1004528)
Supplement: Table S7 — Leukocyte accumulation in the brain. PbA-infected mice with ECM (day 6–8; N = 3), PbA-infected/FTY720-treated mice without neurological signs (day 8 or 9; N = 3), and PyXL-infected mice with HP (N = 3) were vascularly perfused prior to isolation of leukocytes from the cerebrum and cerebellum. Adherent cells were isolated and subjected to flow cytometry. CD45+ F4/80+ cells represent the entire macrophage population of the brain, while the CD45hi F4/80+ macrophages are predominantly blood-derived. The data represent the average cell number per 50,000 events ± STD. Statistical analysis was performed using a 1-way ANOVA followed by Tukey's test for multiple comparisons. See also Figure 3 . (DOCX) [file ppat.1004528.s014.docx]

**Table S7. Leukocyte accumulation in the brain**

|  | **PbA**  **(Day 6-8, N = 3)** | **PbA/FTY720**  **(Day 9, N = 3)** | **PyXL**  **(Day 5, N = 3)** | **PbA vs. PbA/FTY720** | **PbA vs. PyXL** | **PbA/FTY720 vs. PyXL** |
| --- | --- | --- | --- | --- | --- | --- |
| **CD45+ leukocytes** | 23729.3 ± 7573.8 | 6059.7 ± 4070.7 | 4483.0 ± 2971.6 | *P* < 0.05 | *P* < 0.05 | NS |
| **CD8+ T cells** | 829.3 ± 426.1 | 275.0 ± 128.5 | 397.3 ± 224.5 | NS | NS | NS |
| **CD4+ T cells** | 922.7 ± 281.9 | 341.3 ± 187.7 | 589.3 ± 624.6 | NS | NS | NS |
| **Ly6G+ neutrophils** | 1470.7 ± 325.5 | 542.3 ± 279.0 | 518.0 ± 317.2 | *P* < 0.05 | *P* < 0.05 | NS |
| **Ly6C+ monocytes** | 2306.0 ± 1163.6 | 648.7 ± 400.2 | 650.0 ± 587.4 | NS | NS | NS |
| **CD45+ F4/80 (CD11b+) macrophages** | 9648.0 ± 3432.1  (8552.7 ± 3432.1) | 2001.0 ± 1400.9  (2251.3 ± 1698.3) | 938.0 ± 645.9  (1390.3 ± 595.3) | *P* < 0.05 | *P* < 0.01 | NS |
| **CD45^hi^ F4/80+ (CD11b+) macrophages** | 1750.0 ± 285.0  (1600.0 ± 381.5) | 387.7 ± 308.8  (369.3 ± 291.1) | 243.3 ± 171.5  (379.0 ± 281.3) | *P* < 0.01 | *P* < 0.01 | NS |

PbA-infected mice with ECM (day 6-8; N = 3), PbA-infected / FTY720-treated mice without neurological signs (day 8 or 9; N = 3), and PyXL-infected mice with HP (N = 3) were vascularly perfused prior to isolation of leukocytes from the cerebrum and cerebellum. Adherent cells were isolated and subjected to flow cytometry. CD45+ F4/80+ cells represent the entire macrophage population of the brain, while the CD45^hi^ F4/80+ macrophages are predominantly blood-derived. The data represent the average cell number per 50,000 events ± STD. Statistical analysis was performed using a 1-way ANOVA followed by Tukey’s test for multiple comparisons. See also **Figure 3.**
