# Supplementary material for: Experimental Cerebral Malaria Pathogenesis—Hemodynamics at the Blood Brain Barrier
Source: PLoS Pathog. 2014 Dec 4;10(12):e1004528. doi: 10.1371/journal.ppat.1004528 (PMC4256476; doi:10.1371/journal.ppat.1004528)
Supplement: Table S9 — ICAM-1 positive CD45hi and CD45lo leukocyte subsets. PbA-infected mice with ECM (day 6–8; N = 5), PbA-infected/FTY720-treated mice without neurological signs (day 8 or 9; N = 3), and PyXL-infected mice with HP (day 5: N = 3) were vascularly perfused prior to isolation of leukocytes from the cerebrum and cerebellum. Adherent cells were isolated and subjected to flow cytometry. The data represent the average cell number per 50,000 events ± STD. Statistical analysis was performed using a 1-way ANOVA followed by Tukey's test for multiple comparisons. See also Figure 4 . (DOCX) [file ppat.1004528.s016.docx]

**Table S9. ICAM-1 positive CD45^hi^ and CD45^lo^ leukocyte subsets**

|  | **PbA**  **(Day 6-8, N = 3)** | **PbA/FTY720**  **(Day 9, N = 3)** | **PyXL**  **(Day 5, N = 3)** | **PbA vs. PbA/FTY720** | **PbA vs. PyXL** | **PbA/FTY720 vs. PyXL** |
| --- | --- | --- | --- | --- | --- | --- |
| **CD45+** | 8290.3 ± 3551.4 | 2103.3 ± 436.5 | 907.7 ± 126.7 | *P* < 0.01 | *P* < 0.01 | NS |
| **CD45^hi^** | 1161.0 ± 431.9 | 494.3 ± 133.7 | 184.0 ± 73.0 | *P* < 0.05 | *P* < 0.05 | NS |
| **CD45^lo^** | 7129.0 ± 3154.7 | 1608.3 ± 302.4 | 723.7 ± 113.9 | *P* < 0.01 | *P* < 0.01 | NS |

PbA-infected mice with ECM (day 6-8; N = 5), PbA-infected / FTY720-treated mice without neurological signs (day 8 or 9; N = 3), and PyXL-infected mice with HP (day 5: N = 3) were vascularly perfused prior to isolation of leukocytes from the cerebrum and cerebellum. Adherent cells were isolated and subjected to flow cytometry. The data represent the average cell number per 50,000 events ± STD. Statistical analysis was performed using a 1-way ANOVA followed by Tukey’s test for multiple comparisons. See also **Figure 4.**
