# Supplementary material for: Experimental Cerebral Malaria Pathogenesis—Hemodynamics at the Blood Brain Barrier
Source: PLoS Pathog. 2014 Dec 4;10(12):e1004528. doi: 10.1371/journal.ppat.1004528 (PMC4256476; doi:10.1371/journal.ppat.1004528)
Supplement: Table S13 — Endothelial ICAM-1 expression in the cortical microvasculature. Two PbA-infected mice with ECM (day 6–8), 2 PyXL-infected mice with HP (day 5), and 2 uninfected control mice were intravenously inoculated with PE-conjugated rat anti-mouse CD54 and subjected to IVM. Relative fluorescence emission was quantified as described in Materials & Methods and expressed as average ± STD. Statistical analysis was performed using 1-way ANOVA followed by Tukey's test for multiple comparisons. See also Figure 5 . (DOCX) [file ppat.1004528.s020.docx]

**Table S13. Endothelial ICAM-1 expression in the cortical microvasculature**

| **Microvessel** | **PbA Day 6-8, N = 10** | **PyXL Day 5, N = 12** | **Uninfected control, N = 12** | **PbA vs. PyXL** | **PbA vs. control** | **PyXL vs. control** |
| --- | --- | --- | --- | --- | --- | --- |
| **PCV** | 95.2 ± 11.6 | 87.7 ± 13.9 | 23.3 ± 5.2 | NS | *P* < 0.001 | *P* < 0.001 |
| **Arteriole** | 44.4 ± 6.4 | 30.8 ± 2.6 | 14.5 ± 3.2 | *P* < 0.01 | *P* < 0.001 | *P* < 0.001 |

Two PbA-infected mice with ECM (day 6-8), 2 PyXL-infected mice with HP (day 5), and 2 uninfected control mice were intravenously inoculated with PE-conjugated rat anti-mouse CD54 and subjected to IVM. Relative fluorescence emission was quantified as described in Materials & Methods and expressed as average ± STD. Statistical analysis was performed using 1-way ANOVA followed by Tukey’s test for multiple comparisons. See also **Figure 5.**
