# Supplementary material for: Experimental Cerebral Malaria Pathogenesis—Hemodynamics at the Blood Brain Barrier
Source: PLoS Pathog. 2014 Dec 4;10(12):e1004528. doi: 10.1371/journal.ppat.1004528 (PMC4256476; doi:10.1371/journal.ppat.1004528)
Supplement: Table S14 — Number of ICAM-1+ F4/80+ macrophages. PbA-infected mice with ECM (day 6–8; N = 3), PbA-infected/FTY720-treated mice without neurological signs (day 8 or 9; N = 3), and PyXL-infected mice with HP (day 5: N = 3) were vascularly perfused prior to isolation of leukocytes from the cerebrum and cerebellum. Adherent cells were isolated and subjected to flow cytometry. The data represent the average cell number per 50,000 events ± STD. T-test was used to determine significances. See also Figure 6A and 6B . (DOCX) [file ppat.1004528.s021.docx]

**Table S14. Number of ICAM-1+ F4/80+ macrophages**

|  | **PbA**  **(Day 6-8, N = 5)** | **PbA/FTY720**  **(Day 9, N = 3)** | **PyXL**  **(Day 5, N = 3)** | **PbA vs. PbA/FTY720** | **PbA vs. PyXL** | **PbA/FTY720 vs. PyXL** |
| --- | --- | --- | --- | --- | --- | --- |
| **CD45+** | 9306.0 ± 3552.2 | 1834.3 ± 1414.9 | 656.7 ± 504.1 | *P* < 0.01 | *P* < 0.01 | NS |
| **CD45^hi^** | 1713.3 ± 3095.3 | 365.0 ± 298.2 | 219.0 ± 161.6 | *P* < 0.05 | *P* < 0.05 | NS |
| **CD45^lo^** | 7581.7 ± 3636.0 | 1469.3 ± 1122.1 | 435.3 ± 346.7 | *P* < 0.05 | *P* < 0.05 | NS |

PbA-infected mice with ECM (day 6-8; N = 3), PbA-infected / FTY720-treated mice without neurological signs (day 8 or 9; N = 3), and PyXL-infected mice with HP (day 5: N = 3) were vascularly perfused prior to isolation of leukocytes from the cerebrum and cerebellum. Adherent cells were isolated and subjected to flow cytometry. The data represent the average cell number per 50,000 events ± STD. T-test was used to determine significances. See also **Figure 6A and 6B.**
