# Supplementary material for: Experimental Cerebral Malaria Pathogenesis—Hemodynamics at the Blood Brain Barrier
Source: PLoS Pathog. 2014 Dec 4;10(12):e1004528. doi: 10.1371/journal.ppat.1004528 (PMC4256476; doi:10.1371/journal.ppat.1004528)
Supplement: Table S16 — Tight junction protein expression in the cerebral cortex and cerebellum. Cryostat sections of brain tissue from PbA-infected CBA/CaJ mice with ECM (day 6–8; N = 4), PbA-infected and FTY720-treated mice that did not exhibit any neurological signs (day 8 or 9; N = 3), and PyXL-infected mice with HP (day 5; N = 3) were immunolabeled with specific antibodies the TJ proteins claudin-5, occludin, and ZO-1. Confocal microscopy images (3–4 per experimental condition) were acquired under identical conditions. Images were imported into ImageJ for quantification of the average TJ protein-specific fluorescence emission with the threshold set to Triangle. The data represent average fluorescence intensity ± SD in the entire field of observation. Significant differences (* = P<0.05) in TJ protein expression under the different infection and treatment conditions were determined by t-test in relation to uninfected control mice (N = 3). See also Figure S6 and S7. (DOCX) [file ppat.1004528.s023.docx]

**Table S16. Tight junction protein expression in the cerebral cortex and cerebellum.**

|  | **Cerebral cortex** | | | **Cerebellum** | | |
| --- | --- | --- | --- | --- | --- | --- |
|  | **Claudin-5** | **Occludin** | **ZO-1** | **Claudin-5** | **Occludin** | **ZO-1** |
| **PbA / ECM**  **Day 6-8** | 181.6 ± 17.6 | 112.8 ± 22.2 | 45.1 ± 16.2* | 123.7 ± 16.7 | 120.6 ± 20.2 | 75.2 ± 11.9 |
| **PbA / FTY720**  **Day 8-9** | 165.3 ± 44.8 | 114.2 ± 25.3 | 71.0 ± 7.0 | 128.8 ± 62.5 | 109.0 ± 21.7 | 88.3 ± 0.0* |
| **PyXL / HP**  **Day 5** | 148.0 ± 13.5 | 100.7 ± 16.7 | 69.1 ± 12.4 | 132.7 ± 29.3 | 106.5 ± 5.4 | 83.7 ± 2.0* |
| **Uninfected**  **control** | 154.4 ± 33.6 | 114.7 ± 27.3 | 100.4 ± 18.8 | 118.8 ± 26.8 | 96.1 ± 4.5 | 72.0 ± 1.2 |

Cryostat sections of brain tissue from PbA-infected CBA/CaJ mice with ECM (day 6-8; N = 4), PbA-infected and FTY720-treated mice that did not exhibit any neurological signs (day 8 or 9; N = 3), and PyXL-infected mice with HP (day 5; N = 3) were immunolabeled with specific antibodies the TJ proteins claudin-5, occludin, and ZO-1. Confocal microscopy images (3-4 per experimental condition) were acquired under identical conditions. Images were imported into ImageJ for quantification of the average TJ protein-specific fluorescence emission with the threshold set to Triangle. The data represent average fluorescence intensity ± SD in the entire field of observation. Significant differences (* = *P* < 0.05) in TJ protein expression under the different infection and treatment conditions were determined by t-test in relation to uninfected control mice (N = 3). See also **Figure S6** **and** **S7**.
